# Canonical circuit computations for computer vision

**DOI:** 10.1007/s00422-023-00966-9

**Published:** 2023-06-12

**Authors:** Daniel Schmid, Christian Jarvers, Heiko Neumann

**Affiliations:** Institute for Neural Information Processing, Ulm University, James-Franck-Ring, Ulm, 89081 Germany

**Keywords:** Recurrent processing, Feedback, Neural network, Binding, Perceptual grouping, Neuromorphic computing

## Abstract

Advanced computer vision mechanisms have been inspired by neuroscientific findings. However, with the focus on improving benchmark achievements, technical solutions have been shaped by application and engineering constraints. This includes the training of neural networks which led to the development of feature detectors optimally suited to the application domain. However, the limitations of such approaches motivate the need to identify computational principles, or motifs, in biological vision that can enable further foundational advances in machine vision. We propose to utilize structural and functional principles of neural systems that have been largely overlooked. They potentially provide new inspirations for computer vision mechanisms and models. Recurrent feedforward, lateral, and feedback interactions characterize general principles underlying processing in mammals. We derive a formal specification of core computational motifs that utilize these principles. These are combined to define model mechanisms for visual shape and motion processing. We demonstrate how such a framework can be adopted to run on neuromorphic brain-inspired hardware platforms and can be extended to automatically adapt to environment statistics. We argue that the identified principles and their formalization inspires sophisticated computational mechanisms with improved explanatory scope. These and other elaborated, biologically inspired models can be employed to design computer vision solutions for different tasks and they can be used to advance neural network architectures of learning.

## Introduction

The field of computer vision has evolved over decades and was inspired by different concepts and approaches. Simple image processing algorithms are motivated by signal theory (Rosenfeld and Kak 1976) while a normative modeling approach showed that vision can be understood as constrained optimization or optimal statistical inference (Marr 1982; Barrow and Tenenbaum 1978). For example, the task of finding a solution from sparse, discrete, and noisy visual observations lead to regularization approaches solving ill-posed inverse problems (Poggio et al. 1985). Such optimization algorithms share principles of brain-like computation when implemented in a parallel, distributed manner (Poggio and Girosi 1990). The suggested Bayesian mechanisms for perceptual inference were likewise considered as a generic guiding principle for the definition of optimal vision strategies in computational as well as biological systems (Knill et al. 1996). The most recent major shift in focus followed the success of the neural network architecture AlexNet (Krizhevsky et al. 2012) on the *ImageNet* challenge. This started the era of deep learning, in which progress on many computer vision tasks was made by training deep convolutional neural networks (CNNs). While neural networks are clearly inspired by biology (hence the name), most recent breakthroughs are attributed to technical improvements to network components, architectures, and training procedures (LeCun et al. 2015).

Despite impressive advances in the field of computational vision, numerous unresolved problems still remain. For example, state-of-art trained deep network architectures may assign a minimally corrupted input image to a completely different class while a human observer would not even notice the image manipulation (Szegedy et al. 2014). These and other related limitations lead to several recommendations to training procedures, model benchmarking and interpretation of results (Geirhos et al. 2020). Another, more structural deficit is that neural networks are less shape-sensitive than expected. Instead they often use texture information for their final output decision which has been referred to as texture-vs-shape bias (Wichmann et al. 2017; Geirhos et al. 2018; Jarvers and Neumann 2023). Such observations have renewed the motivation to investigate structural and functional principles in biological vision. These biological vision principles make vision robust, flexible, and adaptive in animals, such as primates (Dapello et al. 2020). The goal is to find inspirations that may be used to constrain the layout and function of computational vision architectures, either by their design or by their training.

In this review, we focus on the identification of canonical principles of computation in neural circuits. They will serve as a guide for the integration of processing mechanisms into computational vision models that replicate data from neuroscience and psychophysics and that are capable of successfully processing real-world input data. We review how biological vision can inspire computer vision modeling. This includes a discussion how results from different domains may be analyzed and compared. We sketch canonical principles of computation that provide a rich set of operations which can be deployed for different functions and tasks. The model framework builds upon anatomical and physiological findings, in particular the feedforward, lateral, and feedback information flow in neural networks in the brain. Then, we outline a template for a model architecture composed of computational nodes. These nodes are connected in layers and stacked hierarchically to build complex networks for different computational vision problems. We utilize these components to investigate several computer vision tasks for shape and motion processing. Extensions of the core model components are then shown to be suitable for implementing efficient real-time vision models on neuromorphic hardware. Also we demonstrate how adjusting the efficacy of synaptic weights realizes short-term adaptations to (changing) natural scene statistics.

## Biologically inspired computer vision

### Neuroscience and computer vision—a source of mutual fertilization

The apparent ease and robustness by which biological organisms operate and solve visual tasks motivates taking such functionality as inspiration for computer vision. In recent neural network models, complex detectors are trained end-to-end to generate feature representations at different hierarchical levels. Such deep learning architectures are inspired by the neural architecture of the mammalian visual system. These systems consist of processing stages with increasingly complex response characteristics and feature selectivity (Yamins and DiCarlo 2016). The deep learning architecture AlexNet (Krizhevsky et al. 2012) paved the way for subsequent developments to build trainable networks dedicated to solve computer vision tasks, such as scene recognition, object target detection, image segmentation, or optical flow computation. For an overview see Kreiman (2021). The generic hierarchical structure with a stacked sequence of layers or blocks of core operations transforms ambiguous sensory input into a coherent representation. These representations allow robust decision-making about perceptual categories and control of cognitive or behavioral task output generation. *But do the learned feature detectors tell us anything about neuroscience mechanisms*?

The stages and operations in these networks roughly correspond to the hierarchical layout and operation of cortical (and sub-cortical) stages of the visual system in primates (Cox and Dean 2014). Basic operations mainly focus on local kernel-based convolutions, pooling and sub-sampling, normalization, to name a few. The convolution operations at different stages utilize simple linear weighted summation of activities over small spatial neighborhoods. The resulting scalar amplitude value is then passed through a nonlinear output function. This is analogous to how neurons integrate input activities from presynaptic neurons via their dendrites. The accumulation of superimposed activities that exceed an internal threshold level eventually generate an output activation. This realizes a (generalized) Perceptron as a computational unit (Dayan and Abbott 2001). However, neurons integrate input activity in ways that are more complex than simple Perceptrons (Gerstner et al. 2014). For example, pyramidal cells in cortex integrate information at multiple sites, namely at their basal and apical dendrites, and combine resulting potentials in a nonlinear fashion (Larkum et al. 2018). Körding and König (2001) and Spratling (2002) discuss potential computational roles of segregated integration sites. On a network level, visual processing splits into segregated pathways, which are mainly devoted to processing identity (*What*) and spatial (*Where*) characteristics of the input, respectively (Ungerleider and Haxby 1994). Processing in these pathways is not functionally autonomous but shows cross-pathway interactions (Grossberg 2000). Also feedback recurrency between areas along the processing hierarchy of individual pathways is the rule rather than the exception (Lamme and Roelfsema 2000; Gilbert and Li 2013). On an even coarser scale, the analysis of the overall cortical network graph structure is of interest, in particular its involvement in the activation dynamics. The dynamical processing of sensory input and cognitive state generation during task-related decision-making showed systematic patterns of distributed activations on the connectome (Sporns 2012). So far, such principles have not yet entered routinely in the building of computer vision models.

It is worth noting that objections are being raised questioning the need for biological inspiration to develop computer vision algorithms. Arguments hampering such a dialogue were drawn from differences in research focus and limitations in technology (Cox and Dean 2014). The first argument includes claiming that models in the different domains have different aims. Analyzing cognitive systems and their models at different levels (e.g., adopting Marr’s three levels, Marr 1982) one may conclude that the brain does not use the same general-purpose world descriptions as suggested by computational theory. In addition to such general skepticism, there remain technical arguments against such investigations. This includes the low performance of biologically inspired algorithms executing real-world tasks. Likewise, it is claimed that artificial systems show greater efficiency of particular task solutions in comparison to human vision (see the discussion in Medathati et al. 2016).

### Analyzing vision at a task-level

The opportunity to cross-fertilize between the fields of computer vision and in computational cognitive neuroscience is self-evident. The core question is, *what computer vision can learn from visual neuroscience, and vice versa*? Here, we will focus our discussion on whether and how computer vision models can benefit from ideas derived from insights in neuroscience. Two fundamental questions need to be addressed in such investigations: (i) how to compare model frameworks that were developed in different domain contexts and (ii) how to define a particular task in which computational mechanisms from biology and machine vision are considered.

Computer vision benchmark datasets have been proposed, e.g., the *Middlebury* datasets (Scharstein and Szeliski 2002), for the comparison of model frameworks. The use of benchmarks fosters comparative evaluation of different algorithms. Similarly, the *ImageNet* challenge provides a platform to foster the evolution of deep neural network learning architectures to solve an image categorization task. Building convolutional neural networks (CNNs) that can mimic biological plausibility depends on statistical properties of the training data. For example, the *ImageNet* database is biased toward object categories as they are indexed by the *WordNet* hierarchy (Deng et al. 2009). To balance the apparent bias, Mehrer et al. (2021) curated the *ecoset* data collection that more strongly reflects the occurrence of meaningful objects in human daily life. The evaluation of brain-inspired models against experimental data collected from neuroscientific studies can also benefit from benchmarks. This recently led to the proposal of benchmarks together with scoring metrics, such as *BrainScore 1.0* (Schrimpf et al. 2020). BrainScore’s main purpose is to build a repository of data from a variety of experiments to provide benchmarks for building integrative models of brain-like neural processing mechanisms.

Inspiration from neuroscience for building advanced computer vision models requires the definition of performance properties and functional characteristics that need to be improved and compared against already existing solutions. A simple approach is to evaluate existing computer vision models with respect to their biological plausibility. This can be accomplished by probing them with controlled stimuli using parameter variations as in perceptual psychophysics or neurophysiological experiments. Scoring such models might be challenging since the model design has often been guided by different goal specifications and task definitions. In Tlapale et al. (2010) a first attempt was made to establish a test and evaluation strategy to conduct comparison studies for models of motion computation in biological and computational vision. Further, some of these authors suggest a task-centric framework for relating biological and computer vision in tasks which biological mechanisms face. This includes task definitions and identification of some core challenges in computational vision. Analyzing biological vision mechanisms and understanding their model helps to identify computer vision algorithms with their different tasks and possible solutions. Ultimately, the goal is to identify promising candidates that may serve as a source for developing biological or neuroscience inspired approaches for computational vision problems (Medathati et al. 2016).

### Canonical principles of brain computation

In addition to identifying common goals of computation on a task-level, structural principles of the brain architecture which relate to function are of interest. The focus of this paper is to summarize advances in neuroscience that characterize structural and computational principles in the brain that are relevant for computer vision. The key underlying assumption is that the identified structural properties play a role in the implementation of the computational functions. We highlight canonical principles in brain architecture and function and discuss their relation to architectural principles and computational mechanisms (compare with DiCarlo et al. (2012) and their search for canonical principles in object recognition). These, in turn, might provide inspiration for computer vision algorithms. Following Tsotsos (2014), we suggest to identify such characteristics and to possibly combine such a set of key principles and mechanisms. This may then provide a rich repertoire of operations to build artificial vision systems. We relate such canonical principles and their characteristics to the task-centric approach motivated above. The different modeling examples then suggest cases of biological inspiration and their properties.

The canonical principles that we focus on are summarized in the following. We subsequently provide examples of different modeling investigations and how such principles lead to computational solutions for different visual tasks. These mechanisms provide a basis set of elemental operations with inputs from different feature domains. Different latent representations will be generated for individual tasks and their solutions. The canonical principles considered are the following:

- **Feedforward, lateral, and feedback interaction of activation.** The main sensory-driven information flow is fed through a sequence of stages of sub-cortical and cortical areas. One central feature is that the feature selectivity of neurons that process information at different stages gets progressively more selective. This selectivity leads to optimal input feature compositions. In addition, the receptive field sizes increase along the processing hierarchy. Furthermore, cells selective to specific features receive further input in addition to feedforward driving activity: (i) cells interact laterally over larger intra-areal spatial distances (through horizontal connections) and (ii) they receive input from cells in areas higher up in the processing hierarchy (via feedback). Thus top-down signals are re-entered at representations of lower stages (Lamme and Roelfsema 2000). While such interactions are excitatory in principle, additional input is provided by inhibitory cells. These structural interaction patterns provide the basis for counter-stream information flow in support of binding or grouping related sensory items. Also, recurrences establish specific spatiotemporal encodings, such as temporal oscillations or short-term bursting (Destexhe and Sejnowski 2003; Sherman 2001).
- **Activity normalization and gain control.** Activities in neural representations at various levels of the processing hierarchy can be selectively modulated depending on contextual evidence. The evidence is accumulated at higher levels of the neural processing cascade. Excitatory feedback modulations amplify activities which convey information that match with the top-down predictions (Phillips et al. 2015; Brosch and Neumann 2014a). Normalization via divisive inhibition balances such enhancements. Down-modulation of activation takes place in the local context of a pool of neural responses defined over a space-feature neighborhood (Carandini and Heeger 2012).
- **Cooperative-competitive response integration and segregation.**  Populations of neurons are selectively tuned to individual or multiple feature quantities to build distributed representations of stimulus feature occurrences (Chu et al. 2014). Cooperative mechanisms enable feature binding. Feature binding disambiguates local estimates of feature presence through preattentive grouping. Competitive mechanisms, on the other hand, realize a means of selection and decision-making among different competing alternatives in categorical signal representations (Cocchi et al. 2013).
- **Response adaptation.** Neurons can adjust their responses according to the input statistics and their previous activation (Auerbach and Gritton 2022). Such temporal memory effects lead to short-term adjustment of the overall responsiveness. These adjust the system to achieve optimal and stable perception in a continuously changing environment.
- **Efficient, low-redundancy encoding and processing.** Sparse representation of neural activity encoding from event-based sensing is inspired by the response characteristics of retinal cells (Masland 2001) and extends to thalamic and cortical encoding principles. The key observation is that such encodings provide the basis for efficient low-energy computation in sensory processing. This motivates special sensor principles that are based on address-event representations (AER, Liu and Delbruck (2010)) and reduce the redundancies in transmitted sensory data. Such investigations further extend to neuromorphic computing principles which avoid the von Neumann bottleneck; a bottleneck that is caused by localized processing and data communication of input and calculated output responses (Merolla et al. 2014; Davies et al. 2018).

For each of the above-mentioned principles we provide some example investigations into various tasks of visual information processing. All are based on a small set of key neural processing principles. They demonstrate their capabilities to yield high efficiency through brain-like processing.

**Fig. 1:**
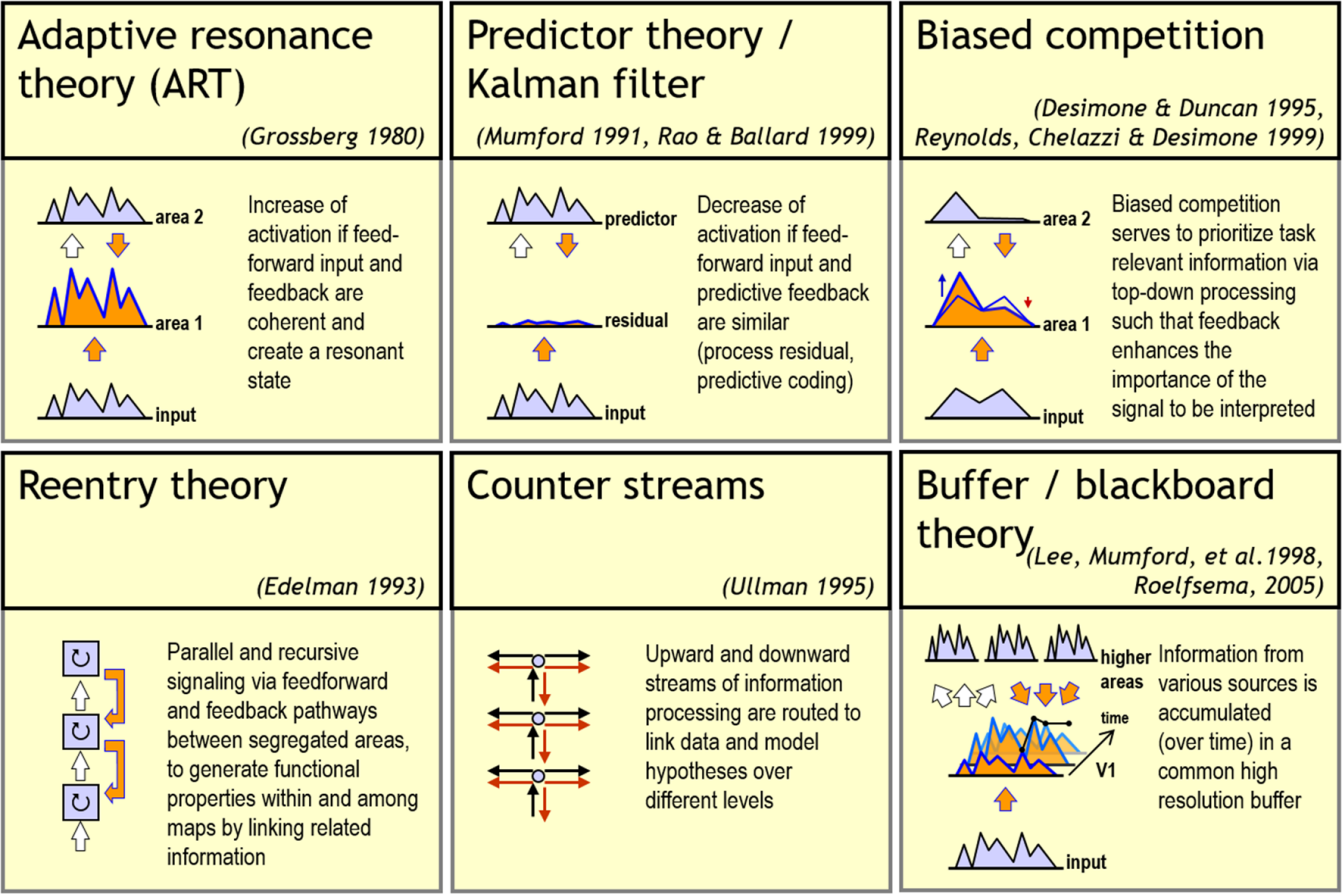
Theories of function of neural architectures and their bottom-up and top-down computation. Over the last decades, different theories about generic principles governing neural circuits and their computations have been described. Explanations of some of the most influential theories are depicted (see individual text boxes for a summary). Common to all these theories is an account for recurrent, feedforward-feedback information processing. The different theories emphasize different properties of recurrent network interaction. Thus, they partially overlap in functionality, share similar principles or are complementary to each other. Sources for each theoretical framework are provided in the list of references

## Model framework

### Insights from neuroscience—counterstreams, re-entry, and resonance

Neural network models can be described at different levels of detail, ranging from molecules over membrane channels, synapses, neurons and networks up to large-scale components interacting at the neural systems level (Churchland et al. 1990). Our focus is at the level of a layered architecture composed of maps of nodes which themselves define groups of neurons of different types. These are connected by weighted links over local spatial neighborhoods. Model architectures consist of a composition of multiple layers where the nodes are connected by weighted directed links to specify the bottom-up feedforward, lateral intra-areal, and top-down feedback signal flow in information processing (Felleman and Van Essen 1991). Information flow from multiple signal streams converge in processing nodes. These nodes combine contextual information with responses that are extracted by localized groups of cells. Such context sensitive integration of information with local feature measurements enables the disambiguation of local responses in a layered representation, and eventually leads to perceptual decision-making.

Several theoretical frameworks have been defined based on anatomical as well as physiological evidence. The frameworks use a set of principles. They realize the overall function underlying the neural network dynamics. In Fig. 1 we present an overview that summarizes different contemporary frameworks which propose specific theoretical accounts of brain computational function. The model framework we advocate in this work comprises a functionality that incorporates different motifs shown in Fig. 1, namely adaptive resonance (ART; Grossberg (1980)), cortical re-entry (Edelman 1993), and biased competition (Desimone and Duncan 1995). Together the key mechanisms allow for integrating information streams. These streams are encoded as neural responses of model cells at a specific location and provide context-reweighted evidence of specific feature occurrences. The neural mechanisms of activation integration are specified in detail below. They include a formal specification of the activation dynamics of the model units.

**Fig. 2:**
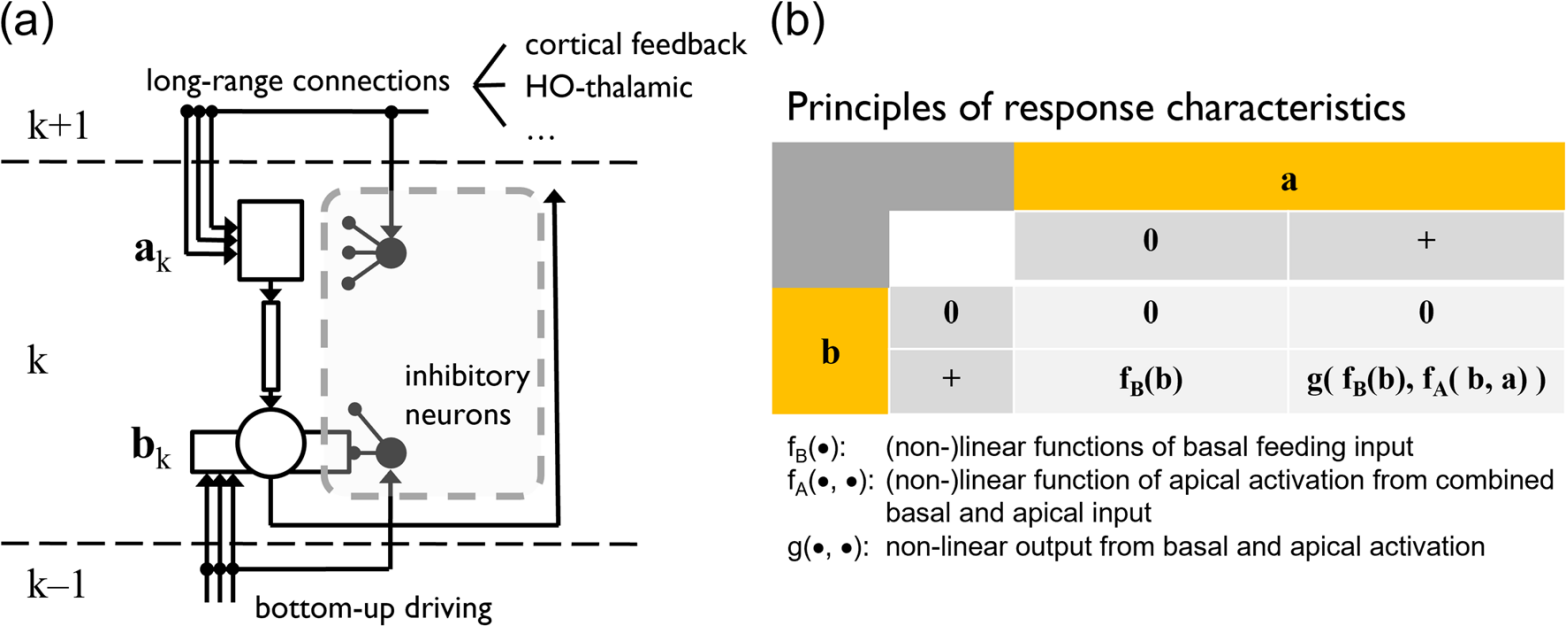
Convergent information streams of bottom-up and top-down information integrated at pyramidal cells in the cortex. **a** A cortical pyramidal neuron at level *k* of the visual hierarchy receives bottom-up driving inputs from lower layers of the hierarchy to its basal compartment $b_k$. In addition, the neuron may receive additional inputs to its apical compartment $a_k$ via long-range connections from various sources. Each compartment integrates its input independent of the other and is potentially gated by inhibitory neurons. The details of the spatial arrangement and mutual interactions between interneurons and their influence on pyramidal cell compartments are omitted here; for more details, see for example Kirchberger et al. (2021). The excitatory inputs to different compartment are nonlinearly combined to generate an output at the soma. For a detailed investigation of the coupling and de-coupling of pyramidal cell compartments, see Suzuki and Larkum (2020). **b** Interactions between basal inputs *b* and apical inputs *a* yield asymmetric response characteristics. The basal input is sufficient to feed the cell generating a response. This response is nonlinearly amplified when apical input is present simultaneously. Such apical input alone, however, is not sufficient to generate a response. The computational logic is summarized in the table

### Integrating feedforward and feedback streams of activity

With the general overview of different theoretical frameworks of cortical function, we identify a major theme of signal integration between different cortical as well as sub-cortical sites by interaction of feedforward and feedback flow of information (Gilbert and Li 2013). Two main questions arise at this stage. The first question asks *At which level are the bottom-up feedforward and top-down feedback streams combined*? In general, such bottom-up and top-down streams converge on common neural substrates. They may be combined at the level of cell populations such that integrated information is shown by the resulting group characteristics. Alternatively, pointwise connections between individual cells may lead to feedforward/feedback integration at the precision of single cells. Physiological experiments provide strong evidence in favor of the latter. Pyramidal cells in cortex integrate feedforward and feedback counterstream signal flows at the level of individual cells and are special in that they have a compartmentalized structure (Larkum 2013). This structure integrates feedforward input at the basal dendritic and peri-somatic region of the cell. In contrast, feedback signals are integrated at the distal apical dendritic site of the cell. While pyramidal cells respond to sensory signals only, they also act as associative devices to link coincident inputs that simultaneously arrive at the different dendritic sites of these cells (Takahashi et al. 2016). Subsequent investigations demonstrate that local *inhibitory* mechanisms at distinct sites of individual cells play a major role in integrating coincident forward and feedback information (Kirchberger et al. 2021). Figure 2a sketches how the main input streams of information converge at different sites of a pyramidal cell in the cortex. In Fig. 2 we only indicate the contribution of inhibitory neurons that are positioned to interact with the excitatory cell compartments. A more detailed specification of the interaction network is beyond the scope of this paper.

The second question details how different signals exert a specific effect on the result of response integration, namely *What is the nature of feedforward and feedback signal interaction*? Ample evidence suggests that in first order interactions in cortico-cortical and in thalamo-cortico-thalamic networks, respectively, feedforward signals act as drivers and feedback signals act as modulators (Guillery and Sherman 2002; Briggs 2020). *Drivers* by definition can generate activations at the cells that integrate the incoming presynaptic input. *Modulators* act to change the amplitude (or gain) of an already existing cell activation. The activation will have been generated by driving input. Consequently, modulating input by itself cannot generate an output activation of a target cell. The compartmentalized structure of pyramidal cells implements such functionality through a net multiplicative effect for coincident basal and apical cell activation (Larkum et al. 2004; Brosch and Neumann 2014b). Thus, loopy network connections composed by asymmetric driver-modulator connections implement a variant of no-strong loops which are less prone to uncontrollable instabilities (Crick and Koch 1998). In Fig. 2b we show the principled response characteristics of a pyramidal cell. We place particular emphasis on the asymmetric roles of basal ($\textbf{b}$) and apical ($\textbf{a}$) input. For a discussion of possible basal-apical dendritic integration functions see Spratling (2002).

### Computational nodes and their neural dynamics

We adopt a mesoscopic level of description of the dynamics of neural responses.^1^ The activity of a node in a network of interacting units represents the state of a neural system, whose temporal dynamics is determined by excitatory driving as well as modulating input and inhibitory activity. A node comprises a spatially discretized group of interacting neurons on a microscopic level. Groups of neurons are described as a neural mass on a mesoscopic level (Grossberg 1988; Breakspear 2017). Such nodes are arranged in sheets or layers of identical units, which are laterally interconnected. The nodes are defined by their input integration and output response calculation. As already outlined above, model architectures consist of multi-layer feedforward streams of information transformation. These are similar to hierarchical signal processing principles in computer vision architectures. In biological vision, feedforward processing is augmented (i) by lateral interaction of feature representations of cells with relatable feature selectivity at different spatial locations in distinct layers and (ii) by feedback activations generated at representations higher up in the processing hierarchy. A node in such a model architecture may consist of pairs of excitatory and inhibitory (E-I) units. These units represent the activation dynamics of a cortical column at different levels of the hierarchy (Wilson and Cowan 1972). Such computational nodes are laterally connected in a layer. They receive external driving and modulating input. However, a node can also range over multiple anatomical layers of subcortical and cortical stages and their intra-node interactions. These nodes are again laterally arranged in a computational layer with grid-like spatial organization (Müller et al. 2020).

**Fig. 3:**
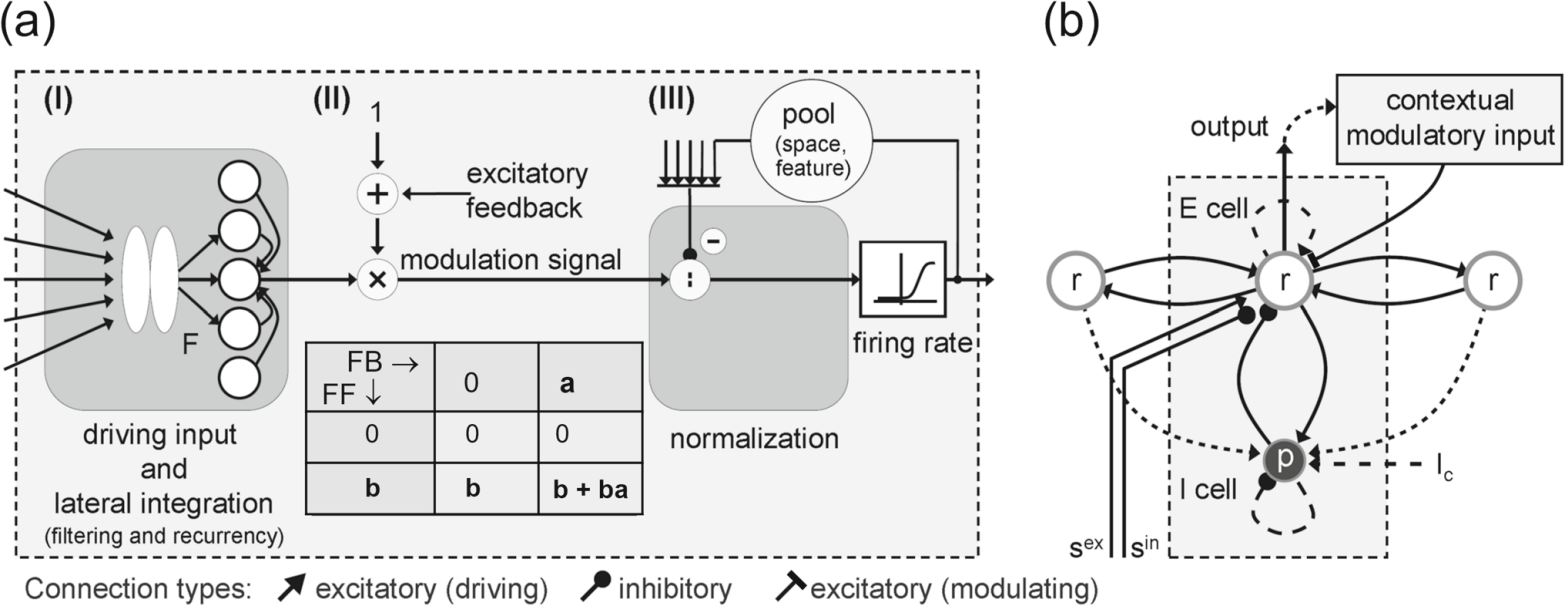
Circuit model architecture for a network node. **a** Three processing elements constitute a node that represents the computation of a cortical column at an abstract level. In the first stage (I) filter responses of input cells generate the driving input to the node (two ellipses symbolize the subfield components of an exemplary filter). These units are laterally coupled in a recurrent field of nodes over a spatial neighborhood (each of those nodes receiving other filter input). The resulting responses define the driving input to the node. This activation is modulated by reentrant signals in the second stage (II). The table at the bottom characterizes the response modulation of feeding input $\textbf{b}$ by reentrant signals $\textbf{a}$. It implements a simplified mechanism of feedforward and feedback integration as shown in Fig. 2b and eqs. 1, 2. The third stage (III) performs a normalization of activation by a pool of neurons. **b** Model architecture in which the three stages are condensed into an E-I circuit. Filtered input feeds an E-node which interacts laterally and is modulated by integrated contextual information. The pool is represented by the I-node. Input lines denote driving signals with excitatory and inhibitory influence (transfer functions are omitted). Each cell’s excitatory activation are enhanced by modulatory FB signals. Spatially arranged cortical columns are shown as laterally connected E-nodes. Each E-cell may incorporate self-excitation (resembling E-E connectivity) and each I-cell self-inhibition (resembling I-I connectivity) shown as dotted lines (sketches adapted from Brosch and Neumann (2014a))

Overall, the sketch of forward and feedback information flow integration emphasizes the local combination of signals at pyramidal cells (Fig. 2a). In case that an apical signal coincides with basal input the output is amplified nonlinearly (Phillips et al. 2016). In Fig. 2b we formalize this algebraically by the response characteristics

1 $$\begin{aligned} r = g\left( f_B\left( \textbf{b} \right) , f_A\left( \textbf{b}, \textbf{a} \right) \right) , \end{aligned}$$

with a nonlinear response function $g\left( \bullet , \bullet \right)$ fed by two arguments defined by the functions $f_B\left( \bullet \right)$ and $f_A\left( \bullet , \bullet \right)$, respectively. The apical function considers the reported signaling between basal and apical sites of pyramidal cells at a more abstract functional level. In Fig. 2, we define a computational unit in a large-scale model composed of neural masses. For clarity, we have simplified the mechanism of local signal integration (Fig. 2a). The function of basal integration $f_{B}$ is chosen as identity, such that $f_{B}\left( \textbf{b} \right) = \textbf{b}$, and the apical function $f_{A}$ implements a coincidence detection mechanism, such that $f_{A}\left( \textbf{b}, \textbf{a} \right) = \lambda \textbf{b} \cdot \textbf{a}$. The function *g* then combines its two arguments additively. The net effect yields a nonlinear output response calculation. In the output, the basal driving feedforward activation gates the modulating feedback activation, thus

2 $$\begin{aligned} act^{FB} = \textbf{b} \cdot \left( 1 + \lambda \textbf{a} \right) . \end{aligned}$$

where $\lambda$ is a scaling constant. This shows that feedforward driving activity is necessary to generate an output activation which cannot be accomplished by modulating feedback activity alone. Coincident activity of forward and backward activation leads to an amplification of the driving input signal by the correlation between the two signals. In order to counterbalance such amplifications in neural responses, a mechanism is required that allows to inhibit or down-modulate activities in a representation. Subtractive mechanisms of neural competition, e.g., for center-surround processing to detect visual contrasts are well-known (Marr and Hildreth 1980). In addition, down-modulation is achieved by a mechanism of activity normalization over a spatial and temporal neighborhood of neurons and their activations (Grossberg 1973; Heeger 1992; Reynolds and Heeger 2009). Feature selectivity of the pooling operation defines a target of investigation. This operation determines the strength of normalization and how interactions can be implemented in a biophysically plausible architecture (Busse et al. 2009; Heeger and Zemlianova 2020).

We combine three different computational mechanisms for a node in a layer of topographically arranged local processors. These components are: (i) input filtering specific to the level in the architecture and recurrent lateral integration of activities generated by neighboring nodes, (ii) the top-down modulation utilizing the mechanism in eq. 2, and (iii) a mechanism for down-modulating activities based on pool normalization (compare with Brosch and Neumann 2014a). A sketch of the components is shown in Fig. 3.

The dynamics of the resulting E-I node is formally described by a pair of ordinary differential equations

3 $$\begin{aligned} \begin{aligned} \tau \dot{r}_{iv} =&-\alpha r_{iv} + \left( \beta - r_{iv} \right) \cdot P\left( F_{iv}, \textbf{r}, \textbf{z}^{FB} \right) \\&- \left( \delta + r_{iv} \right) \cdot \sum _{k} F_{kv} \cdot \Lambda _{ik}^{-} \\&- r_{iv} \cdot g_q\left( q_{iv} \right) ,\\ \tau _q \dot{q}_{iv} =&-\alpha _q q_{iv} + \beta _q \cdot \sum _{kf} r_{kf} \cdot \Lambda _{ikvf}^{pool} + \left( I_c \right) _i \\&- g_q\left( q_{iv} \right) , \end{aligned} \end{aligned}$$

with

4 $$\begin{aligned} \begin{aligned} P\left( F_{iv}, \textbf{r}, \textbf{z}^{FB} \right) =&\left[ F_{iv} + \kappa \sum _{k} r_{kv} \cdot \Lambda _{ik}^{lat} \right] \\&\cdot \left( 1 + \lambda z_{iv}^{FB} \right) . \end{aligned} \end{aligned}$$

Node activities are defined according to a leaky integrator that models membrane potential dynamics with the membrane current as the sum of excitatory, inhibitory, and leak conductances. The excitatory (E) state component *r* integrates the input (right hand side) that is defined by a leak current (first term, with exponential decay rate $\alpha$) and excitatory activation (filter response and lateral integration of node activities, which are amplified by a modulating top-down signal, second term). Inhibitory terms are generated by surround inhibition of filtering (third term) and activation from the pool of neurons defined over a spatial and feature neighborhood for divisive inhibition (normalization, fourth term). The driving forces in the excitatory and inhibitory terms are constrained by the saturation levels, $\beta$ and $\delta$, respectively. Biophysically, these constants relate to reversal potentials of cells. The inhibitory (I) state component *q* integrates the input (right hand side) that is again defined by a leak current (first term) and the weighted integration of E-state activations over a neighborhood in the space-feature domain (second term). In addition, a tonic input level and a self-inhibitory term are optional components (third, fourth term). The finally calculated output activation is determined by the E-state which is passed through a firing rate function *g*. Indexes *i* and *v* (or *k* and *f* in the weighted activity integration) represent spatial locations and quantified feature values, respectively. Different $\Lambda$ kernels denote the weighted connections for activity integration in the feedforward, lateral, and feedback signal pathways, respectively. The modulation of driving input with laterally integrated activity is encapsulated by the function *P*. Here, filtered bottom-up input, intra-layer activity and top-down signals converge at individual sites as depicted in Fig. 3a. The factors $\kappa$ and $\lambda$ denote scaling constants for lateral and feedback activities, respectively.

**Fig. 4:**
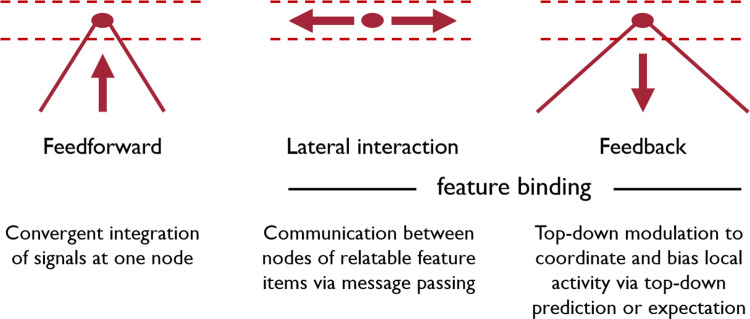
Structural principles underlying feature binding mechanisms. Several principles of feature detection and integration are realized which are based on different structural principles of connectivity and flow of information processing. Feature items are integrated along feedforward signal pathways with convergent information flow (left). Activities that are generated to build a representation in a specific layer communicate their feature-specific activations via lateral connections linking relatable features (middle). Activities of higher-level representations can be re-entered into the response calculations at earlier layers through top-down feedback (right). The cones of convergent bottom-up and divergent top-down information flow share similar properties as in the selective tuning model of attention (Tsotsos et al. 1995). Here, we propose that the influence of the different signal flows is characterized by the different functions of driving and modulating signal interactions

The output calculation of E-I model nodes is more complex than simple filter models used in computer vision or in convolutional neural networks (CNN). Filtering is here part of the response calculation. The activity $F_{iv}$ of the excitatory unit *r* is given in eq. 3. The individual filter kernels are specified in accordance to the function of the processing level in a hierarchically organized architecture. For example, an early filtering stage for detecting contrasts or energy in different frequency channels may resemble isotropic center-surround difference-of-Gaussian filters, derivatives of Gaussians or oriented even and odd symmetric Gabor filters (Szeliski 2010). As pointed out above, standard response functions in layered CNN architectures utilize Perceptron-like computational units with a 2-stage output calculation: (1) weighted summation of convergent input activations^2^, (2) passing the net integrated activity through an output firing rate function. Examples are sigmoidal functions, rectified linear units or hyperbolic tangent functions, depending on the desired functionality within the network architecture.

The response calculation for model units here is more complex and integrates filtering with lateral and feedback mechanisms. At each stage of a hierarchical processing architecture, bottom-up peripheral sensory data streams (filter outputs) are now combined with contextual information. Such contextual information is provided (i) at the same level of the perceptual representations through lateral interaction and (ii) by representations higher up in the hierarchy through top-down feedback signals. In the modeling framework outlined here, the feedback is modulatory and thus amplifies the resulting activations generated by the filtering and the lateral integration (specified in function *P* in eq. 4). Figure 4 sketches the main structural integration mechanisms which accomplish binding of perceptual items.

At the same time, a second modulatory mechanism achieves the normalization of activities over a neighborhood of a current node. Its activation is defined over the spatial as well as the feature domain. The feature domain may consist of different dimensions, e.g., orientation, direction, etc. The neighborhood is often weighted by a function that is separable over the space and feature components. Overall, a pool of nodes is taken into account. The weighted sum of the activations of the nodes in the pool rescales the activation of a target node relative to the overall activity in the pool. Such normalization, or divisive inhibition, is achieved by the last term of eq. 3 driven by $g_q\left( q_{iv} \right)$. The multiplicative scaling of this output by the E-state variable yields a divisive term in the equilibrium response.

In the following, we show in several examples how these computational mechanisms can be utilized to process inputs from different visual domains. We also indicate how such mechanisms relate to algorithmic solutions in computer vision.

**Fig. 5:**
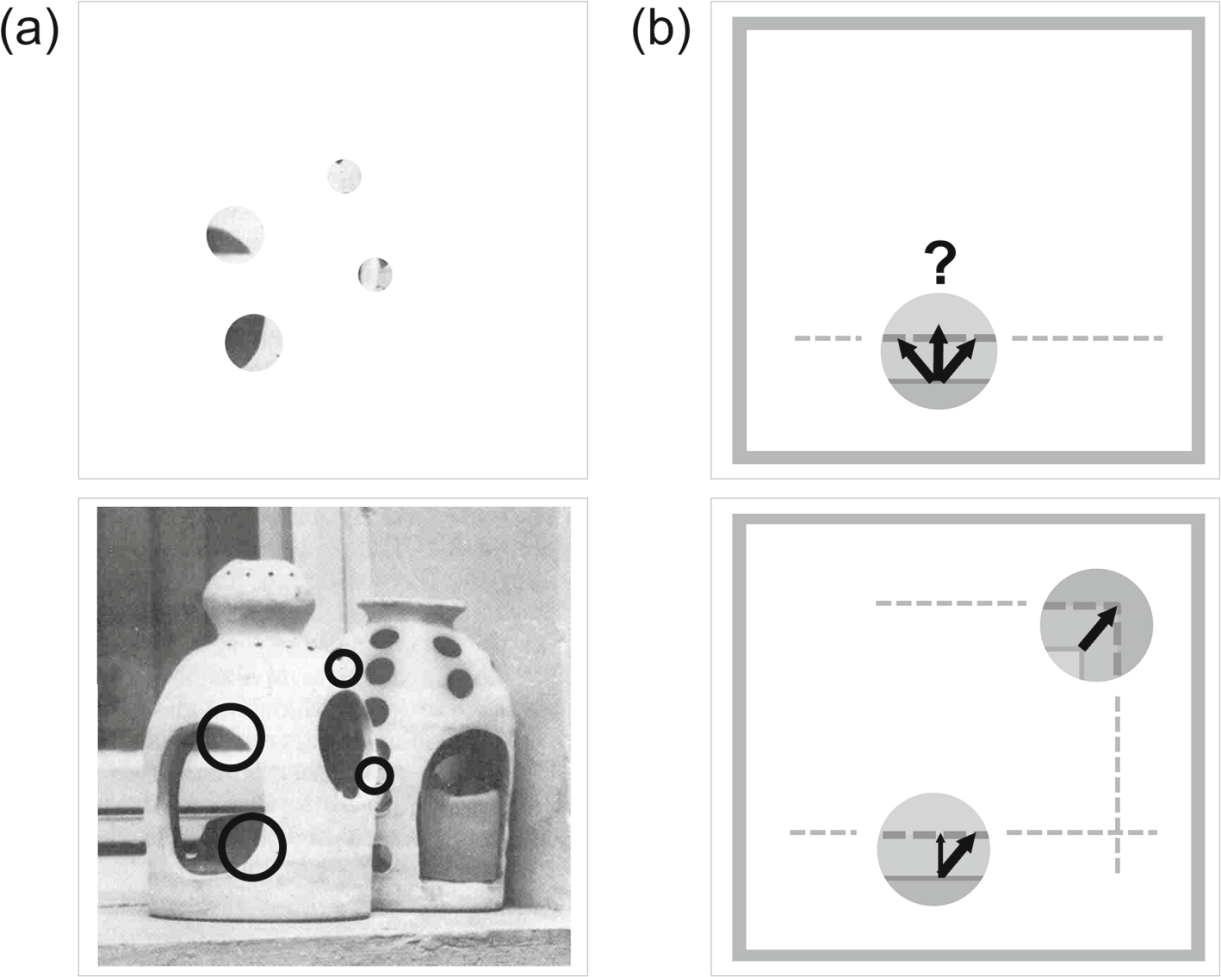
Feature integration and disambiguation for base grouping. **a** A scene with 3D objects seen through circular apertures with different sizes (or scales; top). When the masking patches are removed (bottom) then contextual information signifies the relatability of visual items and provides the basis to disambiguate the local feature characteristics. The circular receptive field shapes are shown to illustrate the position of the apertures in the top image (the photograph of the object scene is reproduced with permission from Peterhans and von der Heydt (1991)). **b** A moving shape is shown as overlay of two temporal snapshots  (contour denoted by solid line at first time point and dashed line at second time point). Top: One part is visible through a single aperture at the bottom of the image. The ambiguity of a single aperture view leads to the perceived normal flow orthogonal to the horizontal boundary (continuous to dashed line). Bottom: If a second aperture at the top right reveals local feature motion which can be combined with the aperture normal flow at the bottom, then the shape appears to coherently move in a direction upward and to the right

## Contour grouping, boundary detection, and texture segregation

### Feature integration and disambiguation to generate base groupings in space and time

The presence of input features is registered by cells with receptive fields which cover only a small spatial input region. The resulting cell responses are noisy and inherently ambiguous. By integrating information gathered from a larger context, this initial evidence can be disambiguated through incremental grouping of visual elements, forming a coherent shape composition (Roelfsema 2006; Elder 2018). The resulting base groupings integrate features over a spatial as well as temporal neighborhood based on their relatability (Kellman and Shipley 1991; Neumann and Mingolla 2001). Such correlations are evaluated based on hardwired connections between basic features. They are computed in parallel at all spatial locations.

In Fig. 5 we show two examples that demonstrate how image structure appears through local apertures, which characterize the neurons’ receptive fields. For each case, a second image is shown with occluding patches removed such that a more global scene composition becomes visible and ambiguities are resolved. The first example (left) shows a static scene of two objects partially occluding each other. Because of the brightness distribution, the silhouette segregating the object in front is completed by filling-in of the illusory boundaries (Peterhans and von der Heydt 1991). Similarly, the second example (right) illustrates a shape moving upwards and to the right which is seen through a single aperture. This leads to perceived normal flow orthogonal to the local contrast (Adelson and Bergen 1985). If the image motion in a second aperture provides additional information about the shape’s motion and assuming that the two apertures belong to the same surface, then the image motion can be disambiguated.

In the following, we illustrate canonical principles summarized in Sect. 2 serve as **biological inspiration** to solve computer vision tasks that require feature integration. The resulting models show **computational properties** useful in computational vision:
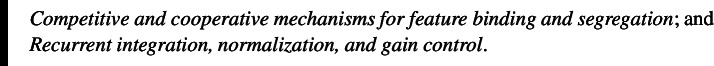


In particular, we show how competitive and cooperative mechanisms implement the perceptual binding of relatable features as well as their segregation into disjoint entities. We show how grouping and competition disambiguate local shape and texture appearances which enables the segregation of salient structure in the spatial domain. In the next section, we focus on the spatiotemporal domain and demonstrate how locally ambiguous image motion is disambiguated and linked to generate coherent representations.

### Contour grouping and texture boundary detection

We use the principles of filtering, integration and modulation outlined in Sect. 3 in two example tasks. The first task is concerned with the context-dependent perceptual binding of local oriented items. Consider the input of a single oriented contrast item that optimally drives a network node. The resulting output activation is compared with the input configuration in contextual displays. The item is surrounded by similar items, with varying orientations, in a spatial arrangement that forms a texture. This leads to a reduction of the cell response at the center location. In contrast, if the bar in the center is augmented by co-axially positioned and like-oriented items, the target cell response increases. This enhanced response even exceeds the reference level for the single input. Using a two-layer architecture, we demonstrate how the computational motifs of filtering, integration, and modulatory feedback can explain such response properties.

The second example task extends the previous one. It requires texture segregation through detection of orientation discontinuities in fields of local oriented items. Here, a multi-layer hierarchical network structure again makes use of successive stages of feedforward filtering which are augmented by feedback connections. Higher levels modulate the responses to detailed texture information at lower stages. Through selective elimination of weighted connections between layers, we demonstrate how feedback enables the network to segregate textured figure outlines from background texture. The main impact is seen in complex cases in which patterns show internal feature contrast.

Both examples refer to the canonical principles listed in Sect. 2 which serve as **biological inspiration** and characterize **computational properties** for computer vision models:
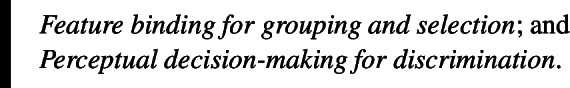


These computational functions are supported by the structural and functional motifs described as building blocks. The specification of the components in each model architecture follow the general structure outlined in eq. 3. We first motivate the logic of investigation to highlight the relevance for task-specific vision. The example results are chosen to demonstrate that dynamic computations achieve the desired results. We relate the test cases and their results to computer vision scenarios and discuss their relevance. For a detailed reference to implementations of the individual cases, we refer to the original publications.

#### Perceptual binding in contour grouping and texture suppression

**Fig. 6:**
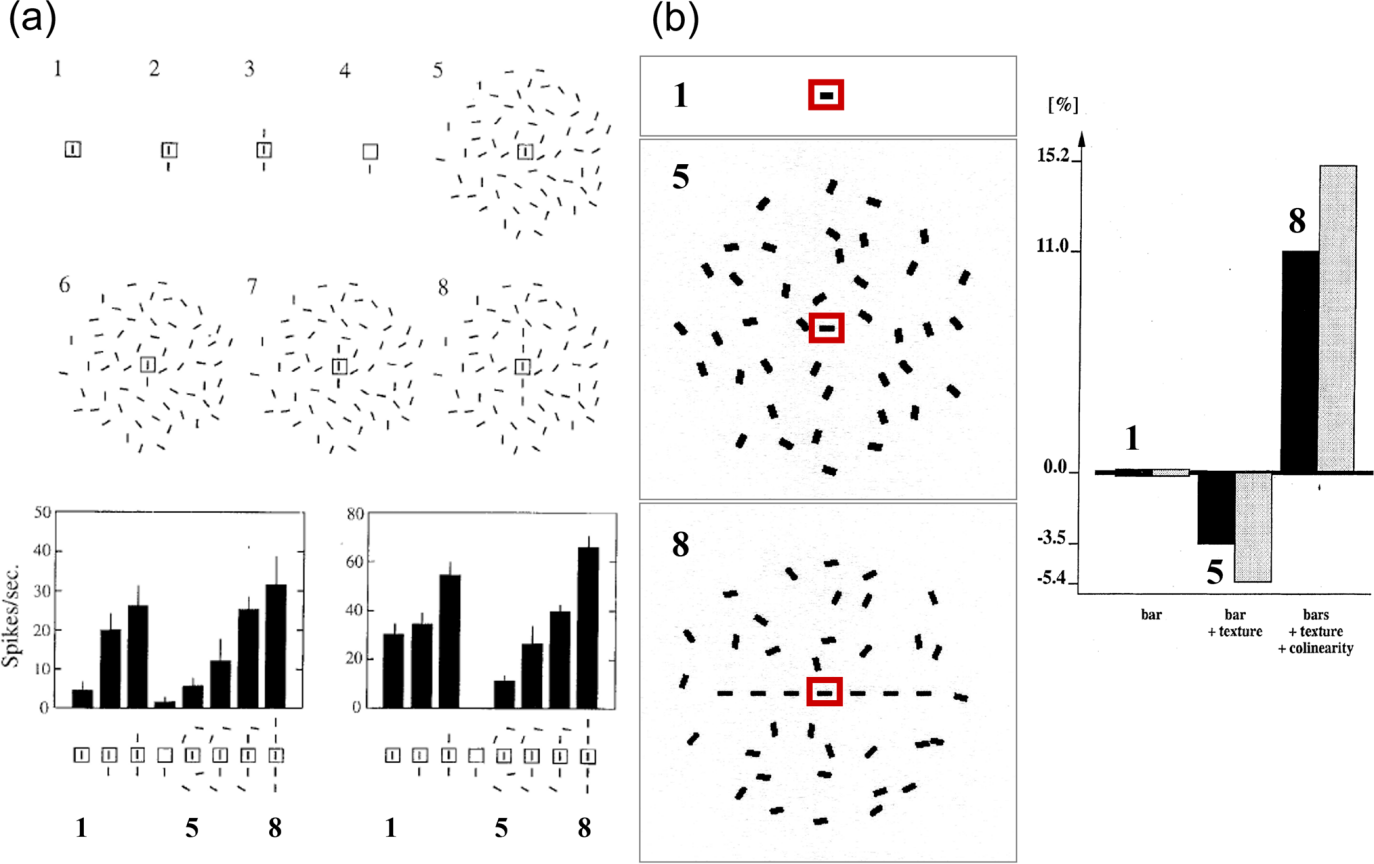
Context-dependent perceptual binding for contour grouping. Neural responses in the early visual cortex depend on the context provided as input to neighboring neurons. **a** Different stimulus configurations are presented (top), while responses of a neuron in a monkey’s early visual cortex are recorded (bottom). A target cell is excited by an optimally oriented bar (configuration 1) that is placed in the receptive field of that cell (RF, square region in the center). The response can be enhanced by additional flanking items placed colinearly outside the RF (configurations 2 and 3) indicating the presence of an extended contour configuration. However, if the central bar item is part of a random texture composed of bars then the response of the target cell is reduced at the center (configuration 5). If the central cell is driven by an optimally oriented bar item, which is again supported by co-aligned flanking bars that form a fragmented continuous contour, then the suppressive effect of the random texture is compensated. The response can even exceed that of the initial oriented input. Configuration 6 to 8 indicate the presence of a perceptual boundary item amidst a cluttered scene (figure reproduced with permission from Kapadia et al. 1995). **b** Neural model simulations reproducing the main effects of neural contour grouping (left) based on inputs that replicate the experimental conditions in (a). The neuron model incorporates computational principles of recurrent interactions by modulatory feedback and pool normalization over a space-feature neighborhood (see main text for details). The response of a target cell that is driven by a single bar item is taken as reference level (anchored at 0). The suppressive effects of embedding the bar as part of a texture and the counter-balancing excitation effects of additional colinear boundary grouping are shown in the bar diagram. Black and gray bars denote two different model parametrizations (figures with adaptations are reproduced with permission from Neumann and Sepp (1999))

This case study investigates how the initial response of a network node to an input stimulus can be modulated. The modulation is achieved by embedding the same individual input item into a larger spatial pattern of similar line items. In Kapadia et al. (1995) an optimally oriented stimulus was placed inside the receptive field (RF) of an orientation-selective target cell. The resulting output activity was used as a reference amplitude. If the input is placed outside the spatial RF, the cell does not respond at all. If additional line segments are placed co-axially to the RF and on its preferred orientation axis, then the target cell increases its response above the reference level. This neural response signals the presence of an extended contour arrangement. The response of the cell is lower than the reference amplitude if the driving input bar is instead part of a texture pattern composed of bars with random orientations. Hence, the cell’s behavior is consistent with the reduced information content of the individual bar in the center of the target cell’s RF, which is now part of the texture pattern. Finally, the oriented bar embedded in the random texture is now augmented by line items placed co-axially to form an extended contour within the texture pattern. The cell response increases again to a level that is larger than the reference level in response to the single item. Overall, depending on the number of collinear line items, the output response can fully compensate the reduction through the texture pattern. The data is shown in Fig. 6 (left column).

The model is implemented as a two-layer hierarchical architecture, with the first layer consisting of cells with oriented RF kernels. The second layer consists of grouping cells, which integrate the responses of first-layer nodes. These processing stages roughly correspond to areas V1 and V2 in the primate visual cortex. Oriented filters in the first layer have even-symmetric weighting functions to sample the orientation space. Responses generated by such filters are inhibited by an activity that is calculated from a pool of cells over the spatial neighborhood and over the orientation domain. The inhibition has a divisive effect, generated by a shunting inhibition mechanism. The resulting output responses are integrated at the next layer by orientation selective cells. The cells integrate their input in proportion to the total coupling strength of oriented feature responses. The weighting over the space-feature domain is visualized as a figure-eight structure of a bilaterally extended integration field that extends along the axis defined by the orientation selectivity of the target cell (compare the taxonomy defined in Neumann and Mingolla 2001). The output responses of cells in this layer are fed back in a topographic fashion to enhance the cell responses of the same orientation selectivity in the previous layer. Details of the mechanisms and their formal definitions can be found in (Neumann and Sepp 1999).

Simulation results of this model are shown in Fig. 6 with the specific input stimulus configurations (middle column) and the resulting relative output responses (right column). First, we calculated the model responses to an oriented bar placed centrally in the receptive field of the target unit. This response serves as reference value to assess the relative change when the input is manipulated. It is assigned to $0\%$ to isolate the change induced by different contexts (right column). We tested two stimuli in accordance with the physiological measures. One stimulus is composed of a texture patch of randomly oriented bar items (texture). The other stimulus is composed of a texture in which the element in the center is augmented by collinear items positioned co-axially to the orientation axis defined by the cell’s RF. Depending on the strength of modulatory feedback, controlled by the parameter $\lambda$, the relative response of the target unit is down-modulated by the influence of the texture pattern in the normalization pool. Through the long-range grouping of collinear items (bound into an extended contour) the response in the second layer is increased due to the increased grouping activity. This signal is sent back to amplify the individual item in the display center. The resulting gain enhancement leads to a compensation of the down-modulation due to the texture-generated normalization. The net effect leads to a further enhancement of the response strength in the center (Fig. 6, right).

Grouping mechanisms that employ Gestalt laws of perceptual organization, such as, good continuation, proximity, and similarity, have been defined in other biologically plausible models (Grossberg and Mingolla 1985; Grossberg et al. 1997; Li 1998) and have already inspired computer vision algorithms for contour enhancement (Parent and Zucker 1989; Guy and Medioni 1996; Hung et al. 2020; White et al. 2022). The architecture outlined here emphasizes that the proposed canonical principles of computation i.e., feedforward and feedback processing combined with competition and normalization, can achieve contextual amplification through grouping, as well as de-amplification or suppression in case of reduced information content of stimulus components. The stimulus features and their configuration can be directly interpreted in light of the predicted output calculations by the architectural components. In addition, the modularity of the model allows composing more complex model variants using the same building blocks of neural computation. An example is introduced and discussed in the next subsection.

#### Texture boundary detection, target contour grouping, and figural texture extraction

The visual system accomplishes texture segmentation by detecting boundaries between homogeneous regions that are composed of texture items. Such boundaries are defined by local feature contrast such as orientation differences, rather than the similarity of the target items (Nothdurft 1991, 1993a). This finding is already interesting since region-oriented segmentation mechanisms in computer vision assume a distance metric between image elements which determines homogeneity in a region (Fu and Mui 1980; Gonzalez and Woods 1993; Szeliski 2010). In various experiments, Nothdurft showed, however, that feature *contrast* characterizes homogeneity for texture as well as for motion and color based segmentation (Nothdurft 1993b). Discontinuities in such changes determine contrasts that delineate homogeneous regions. Boundary detection is not only a prerequisite for region segmentation but also for target detection in attentional visual search tasks (Wolfe 2020).

**Fig. 7:**
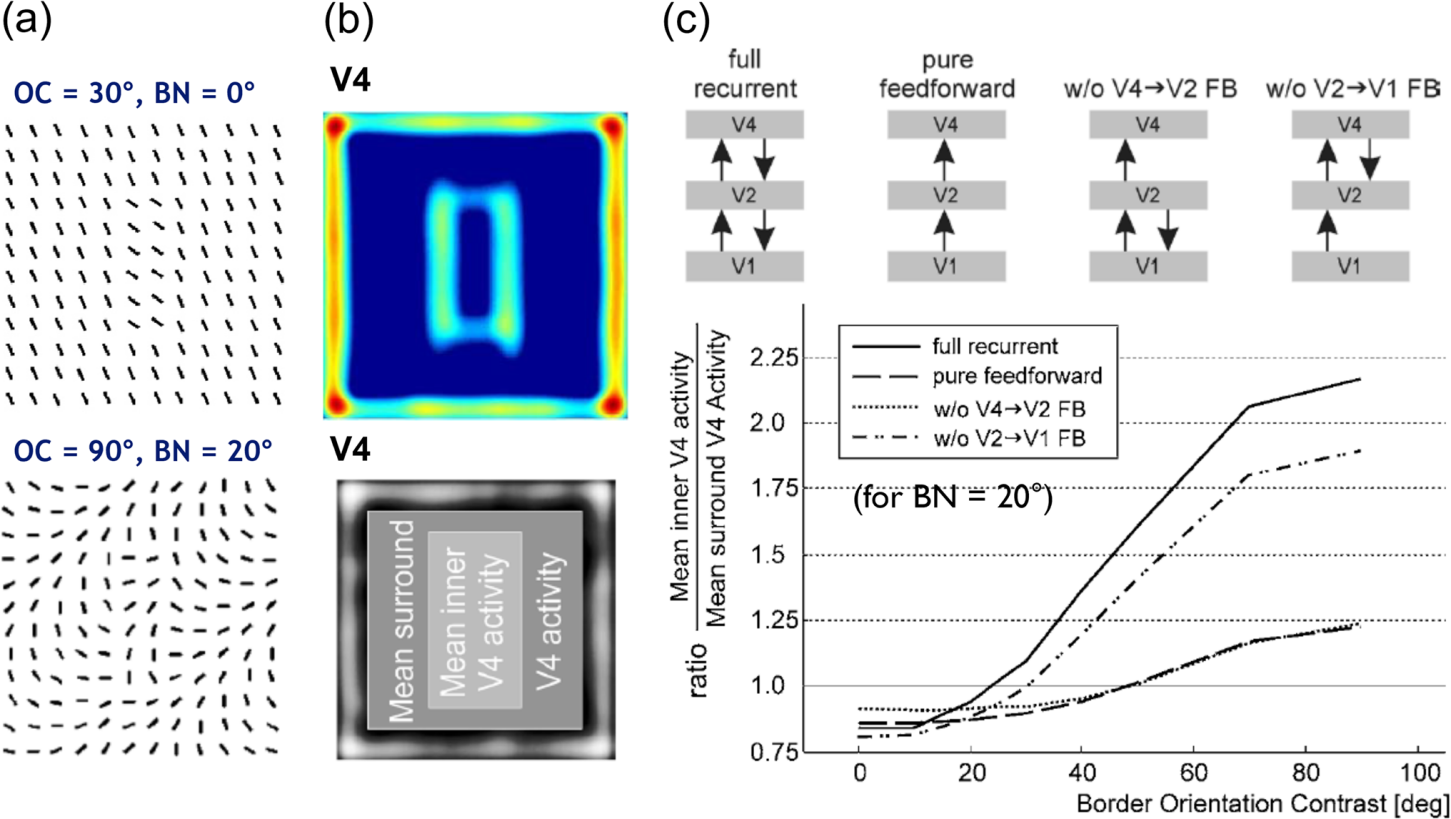
Texture boundary detection and segregation of figure from ground. Panels with texture patterns composed of oriented bars contain a rectangular figure that is either oriented horizontally or vertically. While homogeneity of background and figure is defined by an orientation gradient of the bar items, separation of figure from ground can be established if texture boundaries can be detected from changes in orientation contrasts. In a modelling study, a neural network that utilizes recurrent interactions by modulatory feedback and pool normalization over a space-feature neighborhood has shown to be capable of such texture boundary detection and segregation (see main text for details). **a** Two example displays with different orientation changes within homogeneous regions (BN, background noise) and between homogeneous regions (OC, orientation contrast). OC and BN values determine orientation changes between neighboring bars (in degree). Dashed red rectangles indicate the outline of the figure shape (shown for illustration purposes only). **b** Response of the highest layer of the three-layer network (referred to cortical area V4) for a stimulus defined by ($\text {OC}=30^{\circ }, \text {BN}=0^{\circ }$) (top) and visualization of the activity components used to build a metric for determining the model’s segregation performance (bottom). **c** Model performance (bottom) across different OC values for a given value of $\text {BN}=20^{\circ }$ for the intact network and different model ablations (top, depicted by missing arrows between network layers). The intact model (full recurrent), as well as a variant with missing feedback (FB) from the second to the first layer (w/o V2 $\rightarrow$ V1 FB) reach high performance values once the OC value surpasses the BN value. In contrast, ablations of FB from the third layer to the second layer (w/o V4 $\rightarrow$ V2 FB) or removing all FB connections (pure feedforward) merely result in enhanced activity of figure versus surround. This demonstrates the relevance of higher-level contextual feedback information for texture boundary detection and segregation in cases of noisy cluttered input (figures reproduced with permission from Thielscher and Neumann (2003, 2005))

We extended the model framework for contour grouping in the previous subsection by adding a third block composed of excitatory and inhibitory nodes. All blocks are stacked in a hierarchy and neighboring stages interact via feedforward and feedback connections. Again, feedforward links are driving while feedback connections are modulatory. The three network layers roughly correspond to areas V1, V2, and V4 along the ventral stream in primate visual cortex. While the first two stages have similar operational principles as the model sketched above, the additional layer is composed of a family of oriented contrast filters. These filters possess juxtaposed excitatory and inhibitory subfields of increased size in comparison to the V2 long-range grouping filters. The subfields are selective to different input orientations from the previous processing stages. This makes such nodes selective to orientation contrasts. Details of the mechanisms and their formal definitions can be found in Thielscher and Neumann (2003, 2005).

We demonstrated how different top-down connections contribute to the results of segregating a target figure from the background (see Fig. 7a). In the two depicted cases a vertically oriented rectangular patch needs to be distinguished from a horizontal one. The dotted outline is included for display purposes only and is not shown in real stimuli. The homogeneous textures of figure and background are defined by oriented bar items that are placed on a regular grid with slightly randomized positions and orientations. The region interiors and the boundaries between figure and background have distinct orientation contrasts. In the upper display, the orientation difference between neighboring bar items in figure and background regions is $0^{\circ }$ (BN, background noise). In order to visually segregate the figure patch from the background, an orientation contrast (OC) of $30^{\circ }$ is necessary. A more challenging case is shown in the bottom display. The orientation difference in the homogeneous regions is BN $= 20^{\circ }$. In other words, going horizontally or vertically through the grid of bar items, their orientation changes by the amount of the BN. Under these conditions, in order to detect the rectangular figure patch, the orientation contrast must be increased. In the case shown, we have OC $= 90^{\circ }$.

Using the proposed model architecture, we investigated which network connections contribute most to the desired functionality. To evaluate the strength of contrast selectivity, we defined a metric that compares activations in model area V4, which is selective to texture segregation (Thielscher et al. 2008). We calculated the ratio between mean responses to the figural region (including the boundary) and the mean responses for the background region (Thielscher and Neumann 2007). An example display of model V4 responses and the region masks for calculating the measure are shown in Fig. 7b. For stimuli composed of easy to segregate item arrangements the model architecture successfully segregates the figural patch from the background (results not shown here). However, for more demanding cases in which non-zero OC defines the homogeneous regions, the different stages of processing and their mutual interactions become more important. We investigated four versions of the network architecture with the intact version serving as reference (Fig. 7c, top). We generated lesioned versions by selectively eliminating inter-areal connections. One version reduces to a three-stage feedforward model (eliminating *all* feedback connections) and two variants have the feedback connections between two stages eliminated while the other stays intact. This results in two further versions, namely one with the V4$\rightarrow$V2 connections eliminated and one with the V2$\rightarrow$V1 connections eliminated. We parametrically varied the OC for the texture stimulus with BN = $20^{\circ }$ and probed each version with the respective input stimulus. For each of the processing results we calculated the evaluation measure and displayed the values in the graph in Fig. 7c, bottom. The intact model predicts a smooth increase in the ability to segregate the figural region for increasing OC, which starts to saturate at about $60^{\circ }$. In the lesioned versions of the model, the elimination of reentrant signal flow via feedback leads to diminishing the separability of figural and background region. The version with the V4$\rightarrow$V2 connections left intact only shows minor deprivation in comparison to the other lesioned architectures. This suggests that higher level top-down contextual information makes a major contribution to the performance for the segregation task.

Segmenting a visual scene is a task for generating a meaningful partitioning of the input feature representation into components. In most computer vision approaches the segregation of an input into prototypical parts (characteristic of surfaces or objects) is guided by a coherence or homogeneity property that region elements share. As already highlighted above, Nothdurft’s experiments suggest that such homogeneity properties should include first-order contrast information between feature items as well (Nothdurft 1991). Increased contrasts among feature items, or second-order changes in feature characteristics, are then indicative of boundaries between homogeneous stimulus components. The proposed model, which utilizes the canonical stages of computation outlined in Sect. 3, has been applied to real-world input stimuli with compositions of texture regions. These regions were defined by natural materials or surfaces as documented in the textbook of natural textures and are used as benchmarks in computer vision (Brodatz 1966) (see http://www.ux.uis.no/~tranden/brodatz.html; download 2023). The results demonstrate that computer vision tasks can be solved by such neural mechanisms. They could be further improved by adopting task-specific requirements defined for a given application domain (Bhatt et al. 2007). With such extensions or adaptations, we expect that biologically inspired pixel-wise segmentation mechanisms can be derived and successfully employed in real-world applications.

## Motion detection and integration

### Neural mechanisms of motion detection and integration

We utilize the principles outlined in Sect. 3 and focus now on processes and representations along the dorsal stream in cortex. These investigations show how the canonical principles of context-dependent binding and disambiguation can be applied to processing *spatiotemporal* inputs. We present example tasks of motion processing. One problem focuses on context-dependent binding and disambiguation of local normal flow estimates. Such measures result from the limited spatial information available along extended contrasts, where any shift of luminance structure can only be registered orthogonal to the contrast orientation. This gives rise to the aperture problem of motion (Adelson and Bergen 1985; Watson and Ahumada 1985). This can be solved by integrating multiple local responses of non-collinear normal flows. Different computational strategies have been proposed for such integration of motion estimates, namely vector average, intersection-of-constraints (IOC), or feature tracking (Adelson and Movshon 1982; Bowns 2001).

We use a three-layer neural architecture and apply the neural computational motifs of spatiotemporal detection, integration, and modulatory feedback. Together they can account for local motion prediction and integration. Their action disambiguates local normal flow estimates and, thus, solves the aperture problem. The model makes use of different neural coding strategies, namely rate as well as spike encodings. Velocity (direction, speed) representations at the stage of motion integration in model area MT recurrently interact with motion integration in model MSTl and the local spatiotemporal frequency detection in area V1. This integrates unambiguous feature responses, e.g., at line ends, with ambiguous motion estimates at outlines. The resolution of uncertainty is achieved iteratively and it therefore takes time to disambiguate local estimates starting at localized features. The motion integration robustly combines translatory and rotational motion.

In addition, we demonstrate how such models of motion detection and integration can be implemented on neuromorphic hardware. We show how event-based input from a neuromorphic camera leads to the representation of event clouds (without discrete frames) to be processed by a bank of oriented spatiotemporally sensitive filters. Such a scheme can be executed on a neuromorphic chip to operate in real-time. Additional stages of normalization and the integration in a two-layer bidirectionally coupled network (outlined in the first example) can also be made operational to build neuromorphic schemes of motion analysis. Again, both example cases characterize some canonical principles summarized in Sect. 2 as **biological inspiration** and characterization of **computational properties** to be incorporated in computer vision models:
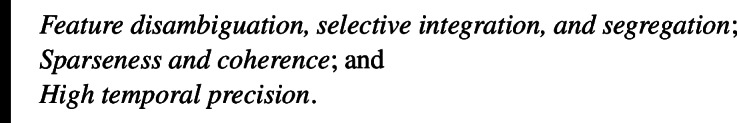


The computational functions are supported by structural and functional motifs from visual neuroscience and can be employed in computer vision tasks.

#### Feature integration and disambiguation for visual motion representation

Local motion seen through an aperture allows measuring normal flow only, leading to the aperture problem. In Pack and Born (2001) it is demonstrated that neurons in the motion sensitive cortical area MT resolve the aperture problem over time. Short oriented bars drive contrast selective cells. MT cells that process the central portion of the bars are initially tuned to the normal flow direction. Within a time window of 60 to 80 ms - depending on the length of the bars - the tuning adapts to encode the physical motion direction, thus solving the aperture problem. This also has direct consequences on the behavior of an active observer who tracks a moving object via smooth pursuit eye movements (SPEM). For example, while fixating the central portion of a backwards tilted bar moving horizontally, the SPEM is initially repelled in the vertical direction. This deviation is proportional to the vertical component of the normal flow for the slanted bar. However, this deflection is compensated such that the SPEM then follows the physical motion in front of the observer (Born et al. 2002). The time for compensation likewise depends on the distance of localized features of the moving shape that have unambiguous image motion.

We proposed a multi-layer hierarchical model architecture of processing along the dorsal pathway with similar components as the model for shape processing in the ventral stream. The model is composed of three layers, which correspond to cortical areas V1, MT, and MSTl. The areas are represented by layers of mutually coupled excitatory and inhibitory cells. In the first layer, we utilize oriented filters with contrast sensitive spatial weighting functions. Their response characteristics are selective to spatiotemporal shifts to the left or to the right of the axis of orientation. Filter responses are inhibited by activity of a pool of cells over a spatial neighborhood, different contrast orientations and spatiotemporal motion selectivities. Like in the model architectures described in the previous sections, the inhibition has a divisive, or shunting, effect. The resulting output responses are integrated by velocity selective cells in the next layer, corresponding to area MT. Cells tuned to different directions integrate input activations from model V1 cells at locations corresponding to the individual speed selectivity of model MT cells. Again, cell responses are normalized by integrated pool activities over a space-velocity neighborhood. Output responses feed forward to a layer of direction sensitive cells, corresponding to area MSTl. They integrate MT output over a large spatial neighborhood and different speed selectivities. Cell responses undergo mutual competition to normalize their activities. All outputs generated at the different model layers also generate modulatory feedback to cells at topographically corresponding positions in the previous layers. The recipient locations are shifted corresponding to the linear forward predictions by motion selective cells. The motion selective cells are able to make these predictions due to their direction as well as speed selectivity. The modulatory enhancement again follows the mechanism specified in eqs. 2 and 4 such that feedback signals are gated by corresponding feedforward activities. Details of the mechanisms and their formal definitions can be found in Bayerl and Neumann (2004); Bouecke et al. (2011); Löhr et al. (2019).

**Fig. 8:**
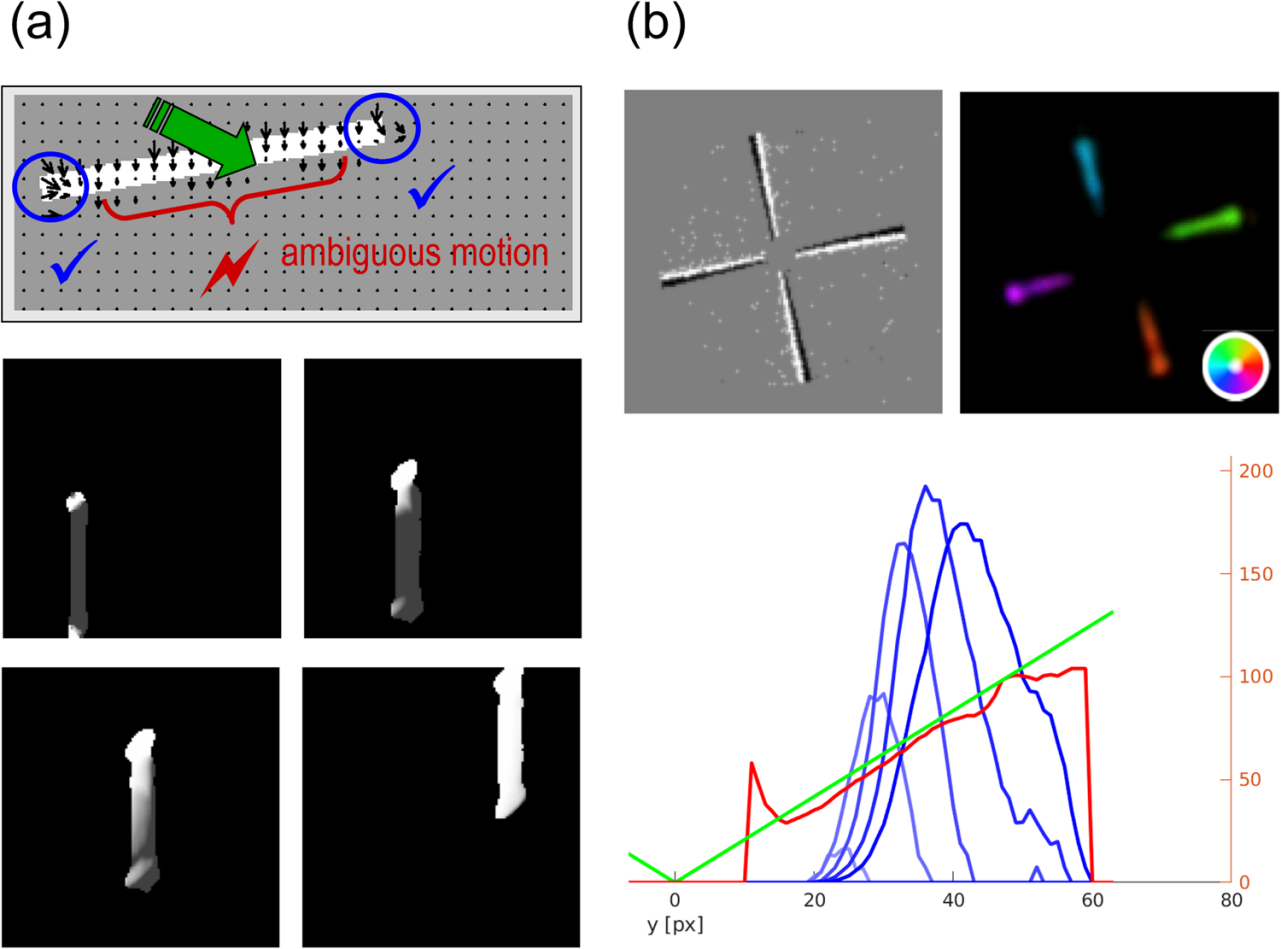
Motion binding and perceptual disambiguation for planar motions. The proposed neural architecture incorporates recurrent interactions by modulatory feedback and pool normalization over a space-feature neighborhood (see main text for details). The function of the architecture successfully infers a wide range of planar motion patterns. **a** Translatory motion of elongated shapes is processed by neurons having only small receptive fields such that only the motion component normal to the outline can be encoded (aperture problem; top (Adelson and Bergen 1985; Watson and Ahumada 1985)). Iterative feedforward-feedback interaction initiates spreading unambiguous feature motion signals at line ends along shape outlines. This disambiguates local motion estimates solving the aperture problem to build a representation of a coherently moving bar (shown is a sequence of four timesteps for a moving bar from bottom left to top right, Löhr et al. (2019); bottom). Brighter values encode smaller average angular error of the motion direction estimate at each pixel. **b** The gradual binding and disambiguation also helps to build representation of rotational object motion. A windmill stimulus with counter-clockwise rotation is represented by different velocities with increasing angular speed as a function of the radius of the position on the arms of the windmill (top). The model framework encodes the motion for different directions (colorwheel) and in different speed sensitive channels (Löhr et al. 2019). Integrating neural responses from a population of neurons with similar direction but different speed selectivities (blue) leads to inference of the shape motion (red). The reference shows the true tangential velocities with their speed gradient as ground truth (green; bottom) (figures reprinted from Löhr et al. (2019) with permission)

Simulation results of the model are shown in Fig. 8. The model predicts that local unambiguous feature motion is integrated at higher stages over a larger spatial scale. This provides evidence for the most likely movement direction which is sent back to enhance the corresponding direction selective responses at earlier stages. Such feedback signals help to disambiguate uncertainties in local motion detection, thus iteratively solving the aperture problem. Such disambiguation of responses follows a grass-fire spreading principle. It starts from localized surface/shape features and propagates inwards along shape outlines. The time needed to disambiguate the normal flow in the center of the moving bar (Fig. 8a) is proportional to the distance that the cooperative-competitive disambiguation wave requires for propagating to the target location. The different phases of the disambiguation are shown for a vertically oriented bar traveling upward and to the right. Feature motion is initially signaled at the line ends with unambiguous motion direction. It then initiates disambiguation until the entire bar is tagged by the same physical image motion direction, thus resolving the aperture problem.

Regularized motion integration schemes in computer vision often employ a data term that minimizes the IOC error of the velocity estimates (Hildreth 1984; Horn and Schunck 1981; Bruhn et al. 2005). In addition to such cases of translatory image motion, we show here the result of motion integration for rotational movement in the image plane (Caplowitz et al. 2007). Figure 8b shows results of processing a windmill shape that rotates counterclockwise around a center point. Motion estimates generated by model MT cells indicate coherent motion for each of the wings (color wheel encoding) accomplished by encoding the increasing angular velocity by speed selective cells. The response characteristics of cells with different speed tuning are shown with the read-out and combination of response amplitudes. The profile (bottom, red curve) shows the reconstructed speed in the image plane together with the ground truth of physical angular motion (green line). This demonstrates that the model architecture is capable to disambiguate local motion estimates and can successfully handle different affine component motions.

**Fig. 9:**
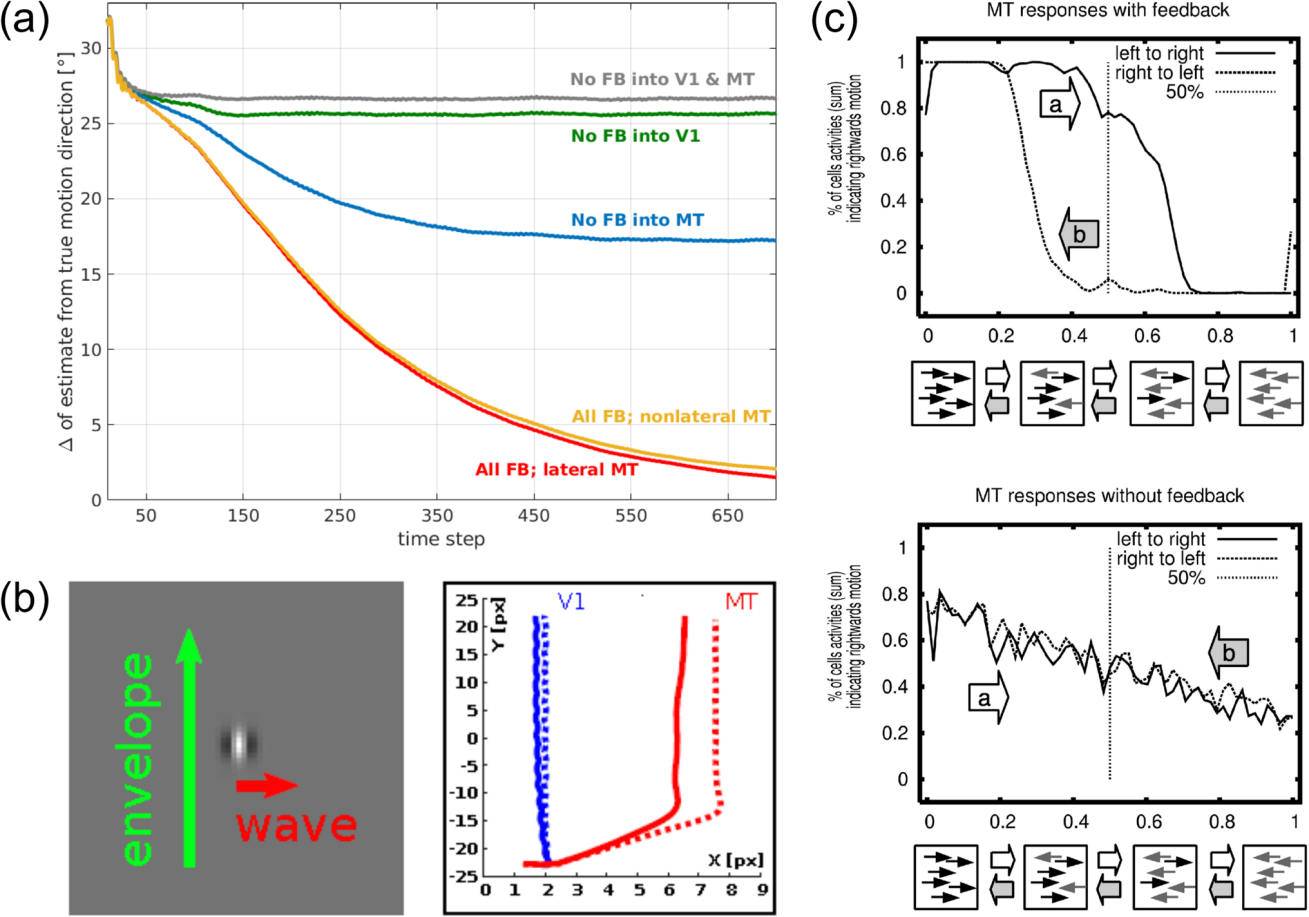
The role of feedback in motion detection and integration. Feedback is shown to play a vital role in neural motion detection and integration. We selectively lesioned feedback connections in a 3-layer model architecture (V1-MT-MSTl) that has been used to process input shown in Fig. 8. The elimination of signal pathways severely degrades performance. **a** We consider the complete model as reference with all feedforward (FF), lateral, and feedback (FB) connections intact. The graph shows the effects of removing FB connections, namely removing FB to the second layer (No FB into MT), removing FB from the second to the first layer (No FB into V1), or removing both FB connections (No FB to V1 & MT). The different graphs show how these manipulations severely impair the network performance through significant increases of the direction error (Löhr et al. 2019) (figure reproduced with permission). **b** In displays of the Curveball illusion component motion of a grating producing a horizontally traveling wave is overlayed by a Gaussian envelope that moves vertically. Together this leads to a perceived diagonally moving object with the orientation depending on the relative velocities of both components (left). The neural model above predicts similar perceived motion trajectories where the representation at the higher stage (MT) showed a displaced estimated motion trajectory akin to the perceptual phenomenon, while the lower stage (V1) more closely followed the true envelope motion of the Curveball object (solid lines, right). If FB from MT to V1 was removed (dashed lines), the deviation of motion prediction became large in MT while the effect in V1 was minute (Schmid et al. 2019). **c** In a random-dot kinematogram (RDK) increasingly more dots of the overall display population change their motion from an initial direction assigned to all dots (i.e., left or right) until all dots move in the opposite direction. Observers watching such motion sequences tend to perceive the same motion direction until an overwhelming number of elements signals the opposite (inlet arrows ”a”, ”b”). Such a hysteresis effect is demonstrated in a two-layer motion model composed of areas V1 and MT (MSTl has been omitted here). The decision is made on the stronger integrated response for the two opposite motion directions integrated over the display panel. The model tracks the motion with a certain momentum before updating the belief state rapidly to accommodate for detecting the opposite motion direction (top). If MT$\rightarrow$V1 FB is lesioned the hysteresis effect is extinguished. Only the relative percentage of motion contribution to either direction is signaled, like in a linear filter (bottom) (panels reprinted with permission from Bayerl and Neumann (2004))

The influence of the modulating top-down feedback at different levels of the model architecture is demonstrated in Fig. 9a. We selectively lesioned the model similarly as in the texture boundary detection model discussed in Sect. 4. The graphs show the consequences of removing single or multiple feedback connections in comparison when all modulatory feedback and lateral cell interactions in MT are intact (red curve). The endpoint error between estimated and true velocity vectors is used as criterion. Removing the lateral connections between MT cells (yellow curve) does not significantly harm the performance. However, a drop in performance is immediately visible when MSTl$\rightarrow$MT feedback is removed (blue curve) and an even stronger effect is seen with the removal of MT$\rightarrow$V1 feedback (green curve). Removal of all feedback connections results in a pure feedforward model, which leads to the worst performance (gray curve).

To illustrate the contributions of modulatory feedback for motion integration and the consequences of their elimination, we show two examples generated by using an intact model architecture and MT$\rightarrow$V1 feedback eliminated. One computational experiment investigates how the model tracks a vertically moving target (envelope of Gabor patch) that captures an internal spatiotemporal horizontal drift motion inside the Gabor carrier (Fig. 9b, left). Both components move with the same speed in the image plane. The model predicts that MT’s population response integrates the component motions from the envelope and the drift of the Gabor waveform (vector summation). The perceived upward motion trajectory which is bent by the lateral offset response leads to the so-called Curveball illusion (Shapiro et al. 2010). The offset in the representation of spatiotemporal motion is small in model V1 with stronger response to the envelope motion, while MT responses are biased towards the perceived motion by the wave-modulation (solid curves). When MT$\rightarrow$V1 feedback is removed, no strong influences on V1 can be observed. The small kernels with spatiotemporal input selectivity measure local changes and only gain weak amplification through MT feedback. In model MT the error is getting larger when feedback is removed (dashed lines). The feedback that biases velocity sensitive representation is missing and the local motion inside the carrier leads to stronger influence on the perceived motion (Fig. 9b, right; for more details, see Schmid et al. 2019).

MT feedback activity generates a perceptual hysteresis effect for prolonged input motion statistics (Fig. 9c). An input sequence is presented that consists of 60 frames each with 60 randomly positioned dots. All dots initially move to the right (random-dot kinematogram, RDK). Each dot has a constant velocity. Over time, in each frame the movement direction of one randomly selected dot changes its movement to the opposite direction. Consequently, after 30 frames 50% dots move to the right and 50% move to the left, while 100% of the dots move to the left at the end of the video. The top-down feedback modulation here further enhances the feature motion that is initially detected by spatiotemporal selective cells. Since MT cells integrate over a larger neighborhood, the feedback generates an inertia locking the top-down motion prediction over a longer temporal period. Initial ambiguity in estimated motion from confounding measures is disambiguated to indicate coherent motion. Integrated motion responses predict the same motion direction even though a linearly increasing number of dots move in the opposite direction. When 60% to 75% of dots have changed their direction the decision becomes unreliable and quickly switches to the opposite direction (Fig. 9c, top; rightward motion sequence ‘a’). The same inertia occurs for opposite motion directions (sequence ’b’), which determines the hysteresis effect. When the modulatory feedback is eliminated, no hysteresis occurs. Now the cell responses indicating rightward (or leftward) motion linearly vary with the relative number of dots in the RDK (for details, see Bayerl and Neumann 2004).

#### Processing multiple motions

The local cooperative-competition mechanisms described above support building representations of shapes moving in the image plane. So far, we discussed how coherent motion interpretations of such objects could be inferred. What about *multiple* object motions in the scene? We briefly summarize some of our investigations that extend the introduced model framework to handle more complex motions that are generated by multiple shapes or patterns and their occlusions.

Multiple objects moving independently in a scene viewed from a specific observer perspective may partially occlude each other with one surface region in front of another more distant surface. Localized features that belong to one surface generate evidence of unambiguous motion, while those generated by multiple surfaces - due to their mutual occlusion - could generate contradicting evidence of feature motion. A classification of terminators has been proposed by Nakayama et al. (1989): an *intrinsic* termination feature belongs to an object or surface, while an *extrinsic* feature belongs to an occluder. The visual system seemingly re-weights the influence of such features to integrate those that belong to a coherent shape and to segregate the occluding segments apart (Braddick 1993). How terminator classification leads to different scenic movement interpretation is demonstrated in the Chopstick illusion (Anstis 1990). Here, two bars forming an X-pattern invisibly occlude each other in their center portion. The two tilted bars move horizontally in opposite directions such that their line ends form intrinsic terminator features. The two segregated bars are perceived to move in opposite directions. Now, two surface regions are placed so that their outline boundaries abut the line ends of the crossing bar at their top and bottom, respectively. These end points are now extrinsic to the bars since they are perceptually owned by the occluding surface patches. As a consequence, the features at the crossing of the bars now dominate the motion feature integration that leads to perceived vertical rigid shape motion (for an illustration, see Neumann et al. 2007). In the Barberpole display, diagonally oriented moving bars seen through an invisible aperture are perceived to move in a direction that is induced by the line ends interpreted as intrinsic to the bars (Wallach 1935). If monocular occlusion cues are added abutting the line ends along parts of the aperture outlines then they are interpreted extrinsic to the moving bars. As a consequence, the pattern motion is biased by those line ends still being interpreted as intrinsic (Liden and Mingolla 1998). In the visual system, such cue-based interpretation is already accomplished at early stages along the cortical dorsal pathway (Pack et al. 2004).

In Beck and Neumann (2010, 2011) the computational model architecture sketched above is extended to integrate motion and shape representations, capitalizing on the complementary mechanisms of segregated motion and shape processing in the dorsal and ventral pathways, respectively (Grossberg 2000). The extended model enhances the integrative function of feature combinations by re-weighting the contribution of shape features dynamically, together with the contour arrangements along the moving shape elements. This leads to context-dependent enhancement or suppression of local features depending on their perceptual interpretation as being intrinsic or extrinsic to the shape and their motion estimation.

Another sort of motion patterns impose a challenge to processing. Consider, for example, object and background motions of a scene that we watch through the window of a train, while at the same time, the movement of people inside the train is reflected in the window. The overlay of the reflection leads to *transparent* motion. Fine structures, e.g., tree branches, leafs or garden fences, partially occlude the background scene. When the finely structured foreground and the background move independently over a large enough spatial region, the patterns are segregated into a percept of *semi-transparent* motion. The movement directions of such overlay patterns must exceed a minimal perceptual angle in order to be segregated into independent transparent motions (Braddick et al. 2002; Treue et al. 2000). Spatiotemporal grouping mechanisms support the perception of transparent motion (Stoner et al. 1990; Braddick and Qian 2001).

We extended the core hierarchical architecture by incorporating a stage with cells selective to large motion patterns and their composition (Raudies and Neumann 2010; Raudies et al. 2011). Model MT cells uniformly integrate motion signals which are then fed forward to model MSTd cells. They selectively integrate local motions which are compatible with large field motion patterns. Model areas are coupled bidirectionally via feedforward and feedback connections to propagate the spatially integrated motion signals. The generic principle of cooperative-competitive processing is realized by the different integration mechanisms in model areas MT and MSTd and their mutual interactions. The model not only replicates physiological as well as psychophysical data, but also makes predictions about the size of coherent motion display patterns required to discriminate transparent from opaque pattern motion (Raudies et al. 2011). The biologically inspired model architecture was then used in a computer vision application to analyze crowd motion in surveillance videos. The task was to detect potentially hazardous situations in the crowd. It was demonstrated that in motion patterns ’dangerous zones’ were tagged when motion transparency and negative speed gradients (decelerated motion) occur simultaneously (Raudies and Neumann 2012).

### Neuromorphic motion detection

The motion processing architecture outlined above involves recurrent computations that are carried out in parallel across the visual field with dynamics that are tuned to capture a wide range of motion speeds, directions, and scales. This combination of parallel processing and precise timing makes it a prime candidate for implementation in neuromorphic hardware and processing of event-based input.

A family of image sensors have been developed that generate address-event representations (AER) instead of recording full-frame images (Lichtsteiner et al. 2008; Posch et al. 2014). This more closely resembles the function of the photoreceptors and successive neural responses in the mammalian retina (Delbruck and Liu 2004). It further supports the canonical principles outlined above, namely *sparseness of representations*, *high temporal precision*, and the *coherence of event-based representation* of spatiotemporal structure. For example, in dynamic vision sensors (DVS) each pixel asynchronously generates a response if the intensity sensed at a pixel position changes significantly^3^. In order to distinguish increases and decreases of intensity, $\text {ON}$ and $\text {OFF}$ event types are represented separately. Individual responses are then signaled as small data packets $\text {ev}\left( \textbf{p}, t \right) = \left( \left( x, y \right) , \text {type}, \text {time-code} \right)$, which uniquely anchor events in space-time. A camera that observes a scene generates clouds of events with events at individual $\left( x, y, t \right)$ locations. This stands in contrast to image frames with dense arrays of pixel values charged over a fixed temporal window (Liu and Delbruck 2010).

Such asynchronously responding cameras yield high temporal response characteristics, which is of utmost interest for real-time vision applications, they can quickly adapt to changing illumination conditions, and they consume only a fraction of power. This has already inspired a community of computer vision researchers to investigate the adaptation of vision algorithms operating on event data. Examples of event-based computer and robot vision are optical flow computation (Benosman et al. 2012, 2014; Orchard and Etienne-Cummings 2014), flow-based navigation (Kashyap et al. 2021), depth from stereo correspondence (Rogister et al. 2011), low-level image feature detection (Clady et al. 2015), and robotic control in a sensory-motor loop (Kaufmann et al. 2019) (see Gallego et al. 2022, for an overview on event-based vision). The development of neuromorphic sensor technology was paralleled by concurrent development of brain-like computing hardware. The hardware frameworks can be distinguished according to the coding of neural responses and their representations, namely analog, digital, and hybrid concepts. At the moment, analog components lack the stability to enable robust programmability and computation. Neuromorphic computing platforms that operate digitally provide the basis for implementing neural sensory algorithms at a larger scale. Examples of such platforms are IBM’s TrueNorth chip (Merolla et al. 2014), SpiNNaker (Furber et al. 2013, 2014), and Intel’s Loihi (Davies et al. 2018, 2021).

**Fig. 10:**
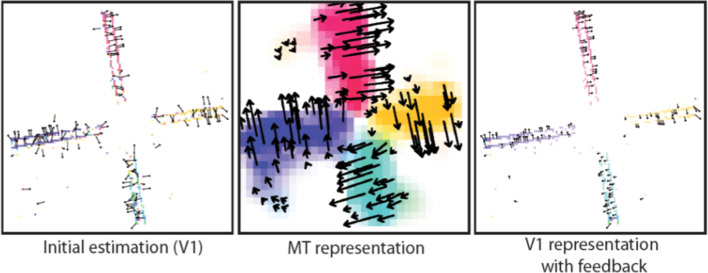
Event-based motion detection and integration. The core mechanisms of the neural model architecture for motion detection (i) allow the processing of camera sensor input that generates address-event representations and (ii) is a candidate for implementing the model on an energy-efficient neuromorphic hardware platform Brosch et al. (2015a). A simplified two-layer (V1, MT) model implementation again has been probed with different types of image motion. For rotational motion (as in the windmill stimulus in Fig. 8) neurons sparsely represent motion at pixels having a spatiotemporal intensity gradient. For rotatory planar motion, as for the presented windmill stimulus, neurons only code for and integrate motion information sparsely at pixels with such space-time gradient in the input. Initial motion estimates in V1 (left) are propagated and integrated on a coarser spatial scale in MT to generate coherent motion estimates (center). These responses are then propagated back to V1 via feedback. In V1 they reduce spurious responses and generate sparsified but coherent representation of spatiotemporal movements along the windmill arms while the background remains silent (panels reproduced with permission from Brosch et al. (2015a))

Neuromorphic vision is a success story for biological inspiration in computer vision in its own right. It has enjoyed increasing popularity in recent years and summarizing all current developments is beyond the scope of this review. However, neuromorphic computing is relevant for our purposes because its sparse, parallel computation and high temporal precision are well suited to implement the canonical computations outlined above. Therefore, we briefly report the adaptation of the first stages of the biologically inspired hierarchical neural architecture which processes event-data acquired by a DVS camera for motion detection and integration described above. We designed a spatiotemporal filter mechanism for estimating the likelihood of local spatiotemporal movements from ON- and OFF-events (Brosch et al. 2015a). The normalization in the competitive stage of divisive inhibition implements a statistical whitening that leads to a broadening of initial direction selectivity for cases of ambiguous motion. A simple read-out mechanism demonstrated the capability of the model as a prerequisite for representing multiple motions, just as required for transparency. The motion detection model has been realized on the brain-like neuromorphic chip TrueNorth that initially filters event-based camera input in real-time (Brosch and Neumann 2015). The more complex neuronal interactions of feedforward and feedback streams have also been realized on TrueNorth (Löhr et al. 2020). A recurrent model mechanism with two hierarchically organized stages of event-based motion detection (model V1) and subsequent large-scale motion integration (model MT) has been realized in (Tschechne et al. 2014; Brosch et al. 2015b). Figure 10 shows some results of processing rotational input motions. The initial responses of model V1 cells are noisy and show spurious responses with significant direction noise, including directions opposite to the true image motion. Such initial responses are integrated by model MT cells with larger kernels operating on a coarser scale. Top-down feedback to the initial filter responses eliminates the spurious responses and enhances the coherent motion responses. Since the recurrent feedback modulates the feeding input activations the spatiotemporal representation in V1 remains sparse.

## Mechanisms of neural adaptation

### Bottom-up and top-down mechanisms in adaptation

In order to detect and encode features under highly variable and uncertain sensing conditions, various mechanisms are required to adapt the gain and response characteristics of the neural system. Such adaptations mainly compensate the neural selectivity for repeatedly presented inputs becoming less responsive. This enables the system to efficiently encode target stimuli and to keep a sensitivity margin to detect and encode novelties in the input. The features of background stimuli and their statistical properties might significantly overlap with those of the target of interest. Thus, additional mechanisms must exist that help to select specific features in a sea of inputs. Attention guides cognitive resources to filter information that is relevant to the current behavioral task while suppressing the remaining stimuli (James 1890). The function of the recurrent feedback network mechanisms in Sects. 4 and 5 increase the gain of layer-dependent filter outputs. They resemble a hierarchical principle of attentive selection. In particular, the combined enhancement (by gain modulation) and suppression (by normalization) realize a type of biased competition to deploy attentive resources to locations and/or features (Desimone 1998). Attention can be guided to a defined stimulus in a top-down observer-driven fashion. The history of the previously selected items subsequently influence the saliency of the items that have not been selected before. They promote effective guidance of attention steering and the selection of target items in everyday scenes and task demands (Vo and Wolfe 2015; Wolfe 2019). Such search tasks rely mainly on episodic memory that guides attention. The neural system utilizes mechanisms to dynamically store and retrieve memory information. It combines them with current input stimuli. Such memory mechanisms span a broad range of temporal scales, ranging from milliseconds to seconds (Tetzlaff et al. 2012). A specific memory trace has been demonstrated in the feedback motion response discussed in Sect. 5. In this example, the history of inferred motion direction re-weights the strength of responses to motion integration. Such trace generates a hysteresis effect for motion directions that are dynamically supported by the recent stimulus history. The hysteresis effect imposes a kind of memory mechanism. It retrieves the dynamic activation and combines it with current input evidence to yield the most likely interpretation of the stimulus.

Both example mechanisms of neural adaptation and selective attention again characterize some canonical principles collected in Sect. 2 as **biological inspiration** and characterization of **computational properties** to be incorporated in computer vision models:
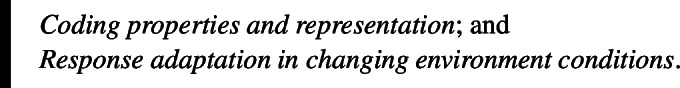


These computational properties are supported by integrating functional mechanisms that extend the model circuit components for recurrent feedforward and feedback interaction between two layers of motion processing (Sect. 5).

### Adaptating feedback loops to natural scene statistics

A key challenge for computer vision algorithms is to remain robust if the statistics of the environment change. Most methods aim to compensate the shift in input statistics, i.e., to shift the inputs back to the original distribution (Schneider et al. 2020). The biologically inspired view suggests a different possibility: neurons continually adapt their selectivity to the current input and their recent response level. In a simple feedforward system, this could lead to unstable learning in which the system loses sensitivity for anything but the most recent experience. However, canonical principles such as the incorporation of feedback and activity normalization can balance stability and plasticity (Grossberg 1980). We exemplify this in the case of motion processing.

Prolonged visual exposure to motion of a particular direction leads to an illusory drift of perceived motion in the opposite direction when the gaze is directed to a still image (motion after effect, MAE). For example, in the waterfall illusion the adapting stimulus is defined by water flowing in one direction (Anstis et al. 1998). The MAE is certainly a cortical phenomenon caused by the selective adaptation of spatiotemporal direction selective cells. In this example, those cells responding to the water reduce their sensitivity by adapting their gain to the continuous flow direction. The adapted cells pay the price of getting a competitive disadvantage during subsequent selection of motion directions when a new stimulus is presented: Motion sensitive cells tuned to the opposite direction now briefly become dominant and signal the illusory flow (Born and Bradley 2005).

We have investigated the functional effects of adaptation at the synaptic level in the neural model of V1 and MT motion processing discussed in Sect. 5. We studied adaptation effects depending on natural scene statistics after skew transformations, which emulate, e.g., the viewing deformations for subjects wearing spectacles. In psychophysical experiments subjects watched short video sequences of natural scenes. These were presented original (un-skewed) or skew-transformed in either upward or downward direction (up-/down-skewed). The skewing transforms significantly change the resulting direction likelihood of motion energies calculated for 12’000 natural image sequences. In three different experimental settings subjects adapted to scene motion statistics for different temporal durations and different repetitions (see the protocol in Habtegiorgis et al. 2019). After such skew exposure (ranging over milliseconds to minutes), subjects had to decide in an alternative forced choice task whether the perceived motion was upward or downward. The research questions of these experiments were: (1) How much the observers were biased by the up- and down-skewing stimulus deformations depending on the adaptation procedure acting over different time-scales; and (2) How a model of cortical motion processing can account for the average subject responses. In order to answer the first question, the overall observers’ responses in the experimental investigations were fitted by psychometric functions using Gaussian regression. Furthermore, the confidence intervals were estimated at the point of subjective equality (PSE). The overall results demonstrate subjects’ time-scale dependent adaptation. The effects of induced bias shifts the PSE in judging the perceived motion direction of the pattern.

**Fig. 11:**
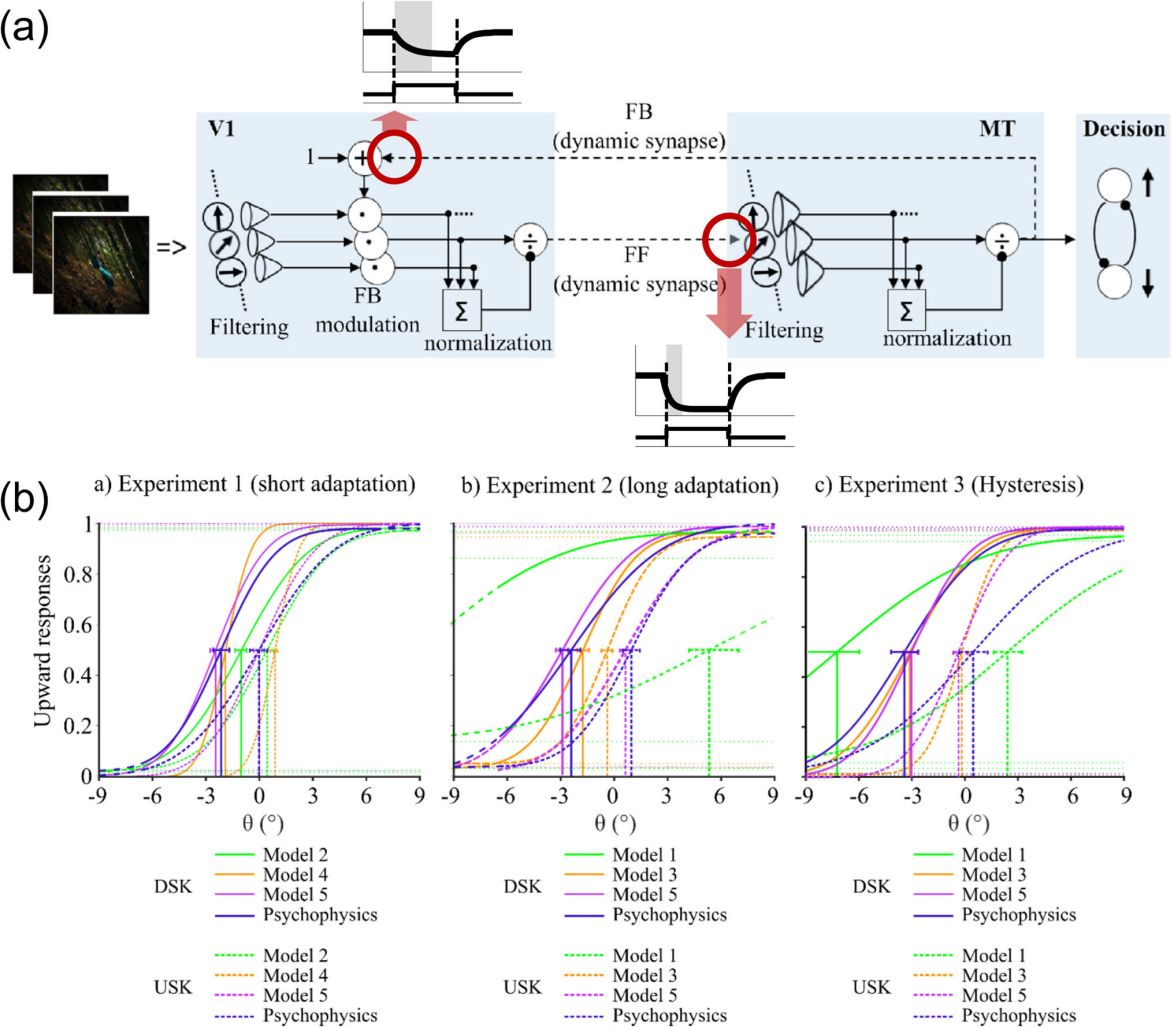
Motion adaptation as a function of input scene statistics. **a** Model architecture with two layers of motion-sensitive cells (V1 and MT) and a final decision layer. The motion-sensitive model areas implement the canonical computations of feedforward filtering, feedback modulation, and pool normalization. The focus of the investigation was on the principle of response adaptation, utilizing dynamic synapses along the feedforward and feedback pathway, respectively (red circles). The strength of connection weights adapted to the input statistics by synaptic vesicle depletion that reduces their transmission efficacy over time. The temporal dynamics of this depletion are shown in the small graphs at the top and bottom. **b** The effect of adaptation to down-skewed (DSK) and up-skewed (USK) image sequences were assessed by probing the response to random dot kinematograms (RDKs). Psychophysical response curves show the ratio of upward responses of human participants and different model variants for different average motion directions $\theta$. Models in which only feedforward synapses were adaptive (models 1 and 2) or in which feedforward and feedback synapses had the same time scale of adaptation (models 3 and 4) did not capture experimental data well. A model that combined fast feedforward and slow feedback adaptation matched experimental results best (model 5; figures are adapted from Habtegiorgis et al. (2019), with permission)

In order to answer the second question, the neural model was adapted to the motion statistics of the skewed natural image sequences. The effects of this adaptation were then probed with random-dot kinematograms (RDKs). Figure 11a shows the neural model architecture with its core processing stages corresponding to areas V1 and MT, respectively, both represented as layers of model cells (Sect. 5). These layers of motion sensitive cells are coupled along bottom-up and top-down signal pathways. Input to the first stage is generated by processing the video input using elaborated correlation-based filters to detect local motion (van Santen and Sperling 1985; Bayerl and Neumann 2004). Output responses of model MT cells generate top-down feedback that is re-entered at the stage of model V1 using the modulatory amplification specified in eq. 4. MT output also drives a final stage of softmax calculation for steady-state decision-making judging the pattern motion direction (Bishop 2006). This selects the maximum of pooled output activations indicating a preference to the upward or downward direction, respectively. Previous investigations have identified that short-term synaptic depression occurs in different brain areas (Abbott et al. 1997; van Rossum et al. 2008). Here, our model of feedforward and feedback cortical motion processing extends the architecture proposed by Bayerl and Neumann (2004). The model provides the basis for assessing the relative contributions of the reciprocally connected streams for the process of adaptation. Different adaptation mechanisms were observed at the cortical circuit level, simultaneously acting at fast and slow time scales (Mesik et al. 2013).

The model makes the prediction that the different observed adaptation time-scales are due to adaptive synaptic efficacy in the feedforward and the feedback streams. In order to assess this prediction, different model variants have been specified, which employ adaptive synapses with slow or fast adapting time-scales. One model architecture extinguishes the feedback connections to test a feedforward model with fast and slow synaptic adaptation (model 1 and 2). The full recurrent model architecture employs adapting synapses either in the forward path (fast time-scale, model 3) or in the backward path (slow time-scale, model 4). Finally, both pathways use adapting synaptic connections with fast feedforward and slow feedback adaptation (model 5). The temporal dynamics of the synaptic efficacy, or weight scaling function, is defined by

5 $$\begin{aligned} \tau \dot{w} = \alpha \cdot \left( 1 - w \right) - \beta w \cdot s(t), \end{aligned}$$

where *w* denotes the scalar weight that is subject to dynamic adaptation (at a rate $\tau$) and *s*(*t*) is the temporal input signal generated by presynaptic nodes. The constant $\alpha$ scales the amount of transmitter production and its inhibition. The constant $\beta$ scales the depletion rate regulating the habituation as a function of the signal strength (Carpenter and Grossberg 1981). Both parameters control the steepness of the weight adaptation and the activity-dependent level of reduced efficacy given by the steady-state effectiveness $w_{eq} = \alpha / \left( \alpha + \beta \cdot s(t) \right)$.

The different models generate predictions for the psychometric PSE using parameter settings ($\alpha$, $\beta$) in the weight scaling function in eq. 5. Only model 5 with adaptive weights along *both* feedforward and feedback pathways responded within the psychophysical range of human performance. The different icons in Fig. 11a symbolize the different signatures of synaptic adaptation. Importantly, fast adaptation along the feedforward and slow adaptation along the feedback path was crucial to decide about the motion directions for all temporal adaptations by human subjects. Figure 11b shows the predictions of the different model variants and the results of human psychophysics for the three experimental adaptation conditions for up- and down-skewing (black). Only model 5 (purple) correctly predicts behavioral adaptation data for all experimental conditions (for details, see Habtegiorgis et al. 2019).

The authors incorporated adaptive synapses in the cortical model architecture of feedforward and feedback motion detection and integration. This specifies a powerful extension that enables the visual representations adapt to the temporal statistics of the input. For computational vision systems, such function defines a candidate mechanism habituating to unforeseen distortions of an imaging sensor. The multiple rates of adaptation in the feedforward and the feedback path, respectively, systematically change the properties of sensing natural environments. The exposure to skewed natural stimuli induces an adaptation in motion perception by changing the weight coefficients in the spatiotemporal kernels.

## Summary and conclusion

### Content presentation and major contributions

In this paper, we have reviewed canonical principles of computation found in biological vision systems that can be utilized to potentially advance computer vision algorithms. These principles of computation were distilled from physiological, anatomical, and behavioral findings. The resulting survey of computational approaches is not exhaustive. Rather, its aim is to distill a conceptual narrative from studies in neuroscience and perception. We do not claim that the principles considered in this work form a complete taxonomy of structural and functional evidence. At the beginning of such a proposal stands the question what computer vision can learn from neuroscience and how the different domains can be compared. Based on our own previous work, we advocate a task perspective on the development and comparison of biologically inspired vision mechanisms.

The primary focus is the integrated processing along feedforward, lateral and feedback streams. Growing evidence supports the view that the recurrent interaction of signal flows determines the flexibility and adaptability of processing at different sub-cortical and cortical stages. Different theoretical frameworks have been developed over the last four decades that predict different computational roles for the feedback recurrence in biological information processing (Fig. 1). Some of the suggested computational principles are similar while others predict a different theoretical goal of computation. However, the theoretical frameworks all share the same generic principle, namely that feedback is essential to implement the hypothesized core computational function. We introduce this summary overview of leading theoretical frameworks and subsequently relate our model mechanisms to elements of the theoretical frameworks. The core element of the modeling and first contribution of the paper is the definition of a computational framework that describes at a mesoscopic level how forward/backward information flow is characterized and how the processing streams are fused at the level of computational nodes.

We emphasized the theme of recurrent feedforward and feedback interaction of information streams. This theme is combined with other computational principles, such as normalization, integration and segregation of responses, and competitive-cooperative mechanisms. We describe an emerging computational motif that is composed of a few core mechanisms to transform an input to an output activity. Crucially, bottom-up signals drive the activity, while top-down signals modulate the activations - in accordance with experimental evidence. The model structure is modular in the sense that it defines a template that can be parameterized. It has then been instantiated at different stages in the processing hierarchy and in the different pathways of form and motion processing. Based on the task perspective we identify and characterize biological computational principles. We define a potential candidate mechanism that is able to solve the considered task and its constraints. We specify only a few characteristic neural computational principles and demonstrate how these operate in a particular task domain. In particular, we specify the basic neural mechanisms for processing shape and form, specifically to demonstrate the binding of local feature characteristics. This leads to the formation of object boundaries and to the segregation of textured regions in cluttered scenes. The same set of mechanisms is subsequently used to investigate the local detection and integration of motion in images. These mechanisms support the disambiguation of local evidences to form coherent representations of object motion. The neural mechanisms robustly handle different types of motion, namely translatory and rotational motion. Together with complementary form information, they can generate early decisions to segregate motion information and assign it to distinct mutually occluding objects in the scene. Cases of illusory motion perception that are predicted by the model are investigated to reveal the detailed computations and properties underlying motion integration.

The computational template can be easily extended to process different representations and to incorporate further functionality. We present examples of adapting the model to process sensor data in an address-event format utilizing neuromorphic computing principles, resulting in efficient, low-redundancy computation with brain-like principles. In addition, we extended the basic model architecture of motion processing to incorporate temporal adaptation mechanisms. These mechanisms adjust the sensitivity of the computational elements when the environmental conditions change. Together, these examples with the specified mechanisms and their example computational demonstrations are further contributions of this paper.

### Task-driven biologically inspired computer vision

We motivated the investigation of biologically inspired computer vision mechanisms and their properties. We set the focus on mechanisms of mid-level vision that operate in the ventral and the dorsal cortical pathway in primates. We emphasized several computational principles, such as recurrent processing, normalization, and gain control, which can be used to explain computational mechanisms of feature binding for grouping and selection based on cooperative-competitive response integration. Assembling them to utilize the complementary characteristics of form and motion, we can realize perceptual decision-making in discrimination tasks and employ coding principles and representation to achieve sparseness, coherence, and high temporal precision. Augmenting the kernel functions with temporal adaptation characteristics incorporates short-term response adaptation to changing environmental properties.

A key behavioral task of vision systems, biological or technical, is object recognition. In DiCarlo et al. (2012) an attempt similar in spirit to this work was made to identify canonical processing principles at different levels of abstraction in core object recognition.^4^ Until recently, the contemporary view was that core recognition can be accomplished by deployment of a serial chain of stacked feature detectors to untangle the object-related identity manifolds (DiCarlo et al. 2012). This principle has been realized in leading biologically inspired models of object recognition, such as Fukushima (1980, 2013); Riesenhuber and Poggio (1999, 2000, 2002); Mutch and Lowe (2008) for instant snapshot recognition and computer vision recognition tasks (Serre et al. 2007). Also hierarchical self-organizing models for spatiotemporal trace-based recognition (Rolls and Milward 2000; Robinson and Rolls 2015) are based on a serial hierarchical feedforward processing chain. Likewise, deep convolutional neural network (DCNN) architectures adopt the same processing structure where each processing node follows the computational logic of a Perceptron with weighted input summation and nonlinear output response, or firing rate, function (Krizhevsky et al. 2012). Some canonical principles similar to those discussed in this article have been employed in such hierarchical recognition schemes, e.g., input filtering, selection, and static divisive normalization via a pool of cells. Only recently, however, recurrent mechanisms along downstream ventral processing have been identified to be crucial to executing object recognition (O’Reilly et al. 2013). Empirical evidence for substantial contributions of recurrent network processing was gathered through the observation of significant shifts in object solution times for input images, which were more challenging to analyze and where feedforward DCNN models showed significant performance drops (Kar et al. 2019). Model architectures have been proposed that incorporate recurrent blocks of processing and are temporally unrolled for the training of parameters for feature detection and integration (Kubilius et al. 2018, 2019). More advanced recurrent schemes have been proposed that incorporate gated units in Nayebi et al. (2018). We suggest that the mechanisms proposed in this review also support advanced object recognition schemes. It has already been demonstrated that a modulatory feedback mechanism can stabilize object category selection in an incremental view-based scheme (Zehender et al. 2003).

This paper discussed modeling examples that were investigated in previous work by our group. As mentioned above, we did not fully elaborate the description relating the selected modeling results to other state-of-the-art neural modeling investigations. The work cited regarding comparable or competing model investigations is selective and certainly incomplete. The research cited served as motivation for the developments shown here, some of which are using similar principles. None of the other models employ the full set of basic mechanisms which, according to our proposal, form a set of canonical principles. The combination of these mechanisms forms a template, which can be adapted to many computer vision tasks. The mechanisms, which define the basic computational motif, were originally utilized to explain or predict data observed in neuroscience or behavioral experiments. However, the models demonstrate their applicability also in processing data from real-world scenarios. The identified canonical principles of neural computation serve as candidate mechanisms for computational vision algorithms and can directly be adapted to recent neuromorphic architectures of brain-like computation.

### Topics not addressed here—possible avenues of further investigation

The focus of our paper was on the specification of neural processes of mid-level vision and their spatio-temporal dynamics. This focus was motivated through a task-level perspective of vision and the assumption of a structure-function relation to understand key neural computational principles. The principles of canonical circuit computation have identified recurrent feedforward and feedback interaction as a main principle of information processing that is employed in the brain. Their closed loop interaction allows biasing the forward sweep of sensory-driven input by contextual information and by decisions made at higher stages in the processing hierarchy. Such interactive processing combines sensory with contextual information and with local mechanisms of competitive processing. These support activity normalization over a specified region in space-feature representations. Together with the canonical feedforward and feedback processes, the mechanisms achieve local decision-making and the assignment of perceptual value to neural activations at a specific stage in the neural hierarchy.

Necessarily, our focus in the discussion leaves out other topics. The following items list major functions in neural architecture that were not considered here, namely

- learning,
- incremental grouping, and
- attention and active perception.

The network functions discussed in this work did not consider any *learning* mechanisms in order to build the necessary filters or feature detectors to form neural representations or adapting them to the input statistics. Major lines of previous investigations capitalize on how neural principles may establish internal neural representations. One such theme investigates how representations self-organize through local correlated activation of coupled neurons and their kernels of local connection weights (Grossberg 1998, 2021). Such local correlative learning is additionally steered by control signals elicited by global modulators so that unsupervised mechanisms are gated by outcomes on the behavioral level of outputs (Kusmierz et al. 2017; Gerstner et al. 2018). The more recent investigation of deep learning architectures has fostered the mutual fertilization of machine learning/artificial intelligence research and neuroscience to build models of hierarchical feature learning and define inductive biases that help identifying generic architectural principles (Richards et al. 2019).

The mechanisms of perceptual grouping considered here process the local feature maps in parallel and automatically. They aim at binding relatable visual items to build coherent representations of boundaries and relatable items as inputs for higher-level processing. Such processing is realized by base grouping operations, which operate by evaluating hard-wired associations in primary feature dimensions. However, evaluating more specific feature elements in the visual input is based on *incremental grouping* operations which act serially (Ullman 1984; Roelfsema 2005). The seamless integration of base and incremental grouping operations builds the perceptual capabilities of flexible reasoning and higher-level control of cognitive tasks.

The serial processing of features and their binding incrementally builds neural representations for task-driven recognition and decision-making. It requires additional mechanisms to schedule and execute processes sequentially (Tsotsos and Kruijne 2014). The selective information capture requires the control via the cognitive mechanisms of *attention*. Attentive selection enables an observer to actively pick relevant information from the wealth of input streams while at the same time discarding or filtering other information (Tsotsos et al. 1995). Processes underlying attention mechanisms can be deployed to actively select features at specific locations, to select specific stimulus characteristics, or to tag a perceptual object of arbitrary size. Such function shows that mechanisms of parallel as well as serial grouping and selective attention are closely intertwined (Roelfsema 2006). The functional integration of grouping and attention mechanisms defines the basis for advanced models of active perception (Bajcsy et al. 2018).

All such processes and mechanisms discussed in the paragraphs above, define possible routes to further investigate the presented canonical circuit mechanisms of brain computations and their employment in approaches to computer vision. Tasks related to these functionalities define challenges for investigations to utilize the discussed network functions and how they might be integrated in new more elaborated principles of computation and learning. We suggest that these are candidates to provide an enriched repertoire of brain-inspired mechanisms, that define computational motifs to develop future systems level computer vision mechanisms.

## Data Availability

Not applicable.

## Footnotes

1 The nervous system contains structures and processes on a range of different spatial resolutions, from molecules over neurons to networks and interacting neural systems (Churchland et al. 1990). Similar to other sciences, such as physics, different levels interact, but can be decoupled for the sake of functional analysis (Anderson 1972; Fannes et al. 1994; Buzsaki and Christen 2016). At the *microscopic* scale, models describe neuronal activation on a sub-cellular level, e.g., individual ion channels. Such models are too complex to investigate functions at the level of interacting neural subsystems in cortical areas, which are instead characterized at a *macroscopic* level of analysis. Analyses at the level of groups of neurons, receptive fields, and small networks range at a scale between the micro- and macroscopic level. This is called the *mesoscopic* scale of resolution (Fregnac et al. 2016). We focus here on this level, which allows us to link neuronal computational principles with function at a behavioral level (Carandini 2012).

2 Dot product of input activation and weight kernel. An additional bias term is incorporated which serves as a threshold for the output function. It defines an extra dimension in the weight kernel.

3 Pixels may interact with other pixels in a local spatial surround, depending on the device parameter settings.

4 The rapid discrimination of a single target object from other objects in cluttered scenes and without any cuing information has been coined *core* object recognition (DiCarlo and Cox 2007).
